# The neuronal chromatin landscape in adult schizophrenia brains is linked to early fetal development

**DOI:** 10.21203/rs.3.rs-3393581/v1

**Published:** 2023-10-02

**Authors:** Kiran Girdhar, Jaroslav Bendl, Andrew Baumgartner, Karen Therrien, Sanan Venkatesh, Deepika Mathur, Pengfei Dong, Samir Rahman, Steven P. Kleopoulos, Ruth Misir, Sarah M. Reach, Pavan K. Auluck, Stefano Marenco, David A. Lewis, Vahram Haroutunian, Cory Funk, Georgios Voloudakis, Gabriel E. Hoffman, John F. Fullard, Panos Roussos

**Affiliations:** 1 Center for Disease Neurogenomics, Icahn School of Medicine at Mount Sinai, New York, NY 10029, USA; 2 Friedman Brain Institute, Icahn School of Medicine at Mount Sinai, New York, NY 10029, USA; 3 Icahn Institute for Data Science and Genomic Technology, Icahn School of Medicine at Mount Sinai, New York, NY 10029, USA; 4 Department of Psychiatry, Icahn School of Medicine at Mount Sinai, New York, NY 10029, USA; 5 Department of Genetics and Genomic Science, Icahn School of Medicine at Mount Sinai, New York, NY 10029, USA; 6 Institute for Systems Biology, Seattle, WA, 98109, USA; 7 Mental Illness Research Education and Clinical Center (MIRECC), James J. Peters VA Medical Center, Bronx, New York, 10468, USA; 8 Human Brain Collection Core, National Institute of Mental Health-Intramural Research Program, Bethesda, MD, USA; 9 Translational Neuroscience Program, Department of Psychiatry, University of Pittsburgh School of Medicine, Pittsburgh, PA, USA

## Abstract

Non-coding variants increase risk of neuropsychiatric disease. However, our understanding of the cell-type specific role of the non-coding genome in disease is incomplete. We performed population scale (N=1,393) chromatin accessibility profiling of neurons and non-neurons from two neocortical brain regions: the anterior cingulate cortex and dorsolateral prefrontal cortex. Across both regions, we observed notable differences in neuronal chromatin accessibility between schizophrenia cases and controls. A per-sample disease pseudotime was positively associated with genetic liability for schizophrenia. Organizing chromatin into *cis*- and *trans*-regulatory domains, identified a prominent neuronal *trans*-regulatory domain (TRD1) active in immature glutamatergic neurons during fetal development. Polygenic risk score analysis using genetic variants within chromatin accessibility of TRD1 successfully predicted susceptibility to schizophrenia in the Million Veteran Program cohort. Overall, we present the most extensive resource to date of chromatin accessibility in the human cortex, yielding insights into the cell-type specific etiology of schizophrenia.

## Introduction

The epigenome encompasses the architecture of DNA packaged inside the nucleus, including chromatin accessibility, DNA methylation and a range of histone marks. This orchestration dynamically regulates gene expression throughout life. Marked by rapid and intricate changes, the early stages of brain development offer a critical window where genetic and environmental factors converge, ultimately influencing neural circuitry and pathways that shape behavior and cognition (1–3). The interplay between genetics and environment (GxE), plays also a pivotal role in neurodevelopmental disorders, including schizophrenia (4). Within this highly complex landscape, the epigenome emerges as a landmark for navigating the link between genetics, environment, and schizophrenia. Epigenetic marks not only reflect the combined influences of GxE, but also hold the potential to be modified by therapeutic interventions, offering new avenues for treatment strategies.

Investigation of the epigenetic architecture of schizophrenia using high-throughput assays such as the assay for transposase-accessible chromatin followed by sequencing (ATAC-seq) and chromatin immunoprecipitation sequencing (ChIP-seq) has been limited to two main studies. In the first study, ATAC-seq was applied in the homogenate prefrontal cortex (PFC) of 135 schizophrenia cases and 137 controls (5). In the second study, ChIP-seq for H3K27ac and H3K4me3 was performed on PFC neurons from more than ~120 schizophrenia cases and a similar number of matched controls (6). Despite these valuable resources, these studies have certain limitations. Firstly, the representation of cell- and region-specific open chromatin regions (OCRs) is absent. Also, the disease specific chromatin landscape that encompasses all regulatory elements is incomplete as the markers used, H3K27ac and H3K4me3, only identify active enhancers and promoters.

We aimed to address these limitations by profiling, via ATAC-seq, cell- and brain-region-specific chromatin accessibility patterns in postmortem brain samples from 469 unique donors, comprising controls and individuals diagnosed with schizophrenia and bipolar disorder (BD). Fluorescence Activated Nuclei Sorting (FANS) was used to isolate neuronal and non-neuronal nuclei from two disease relevant brain regions: the PFC and the anterior cingulate cortex (ACC), creating the largest collection, to-date, of disease associated chromatin accessibility profiles in the human brain. We used this dataset to examine specific patterns of chromatin organization in different disease contexts. We focused on schizophrenia and interrogated the higher order chromatin structure spanned by *cis*- and *trans*-regulatory domains (CRDs and TRDs, respectively). Integration with chromatin accessibility from the human fetal cortex annotated the spatiotemporal patterns of schizophrenia-associated OCRs in TRDs. Finally, stratification of polygenic risk scores (PRS) using genetic variants within TRDs identified the functional sub-structure of higher order chromatin that predicts schizophrenia cases in an independent cohort from the Million Veteran Program. Due to the absence of strong findings associated with BD, we limited our downstream analysis to schizophrenia and controls.

## Results

### Population-scale chromatin accessibility data captures cell type-specific enhancer-promoter interactions in human cortical brain regions

**Figure 1:**
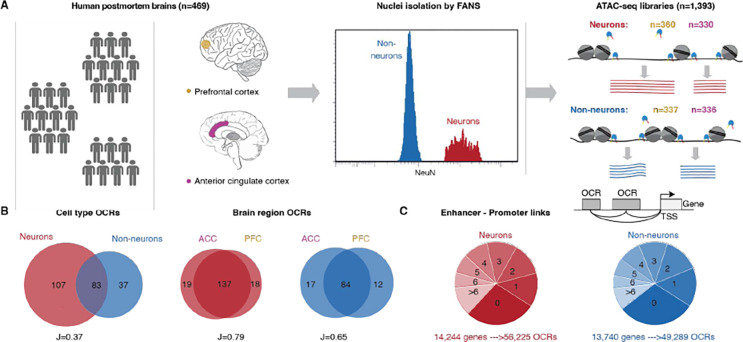
Population-scale chromatin accessibility analysis in the human brain. **A**) Brain tissue specimens were obtained from 469 unique donors, comprising individuals with schizophrenia (SCZ) (n=157), BD (n=77) and controls (n=235). Neuronal and non-neuronal nuclei were isolated by FANS and ATAC-seq profiling was performed to generate a total of 1,393 libraries. **B**) Venn diagram showing cell-type (left) and brain region (right) specificity of identified OCRs. **C**) Top: Schematic to show enhancer-promoter links. Grey box, light grey box and black arrow represent OCRs, TSS and gene body, respectively. Bottom: the distribution in pie charts show stratification of 19,749 genes annotated to the number of neuronal (shades of red) and non-neuronal (shades of blue) OCRs.

To study the relevance of non-coding genomic regions to serious mental illness, we performed ATAC-seq on neuronal and non-neuronal nuclei in frozen postmortem tissues from PFC and ACC in a cohort of schizophrenia (n=157), BD (n=77) and controls (n=235) (Fig. 1A, Fig. S1 and Table S1A–E). After quality control measures, we obtained a total of 1,393 libraries, collectively consisting of over 54.8 billion unique reads (Fig. S2–S3, Table S1F, and methods). Given that the majority of variance in OCR expression was attributed to cell type (Fig. S4) and considering the substantial differences in cell-type specific OCRs (Fig. 1B), along with significant overlap across cortical region specific OCRs, we examined neuronal and non-neuronal samples separately in subsequent downstream analysis. A total of 391,420 neuronal and 260,431 non-neuronal OCRs were identified, of which, the majority were distributed outside the transcription start site (TSS) (Fig. S5A, see data and materials availability). Over 83% of the OCRs identified in this study overlapped with previously observed regulatory elements from reference studies (5, 7–12) (Fig. S5D), and the overlap was higher for matched cell types (Fig. S5E) and known cell type markers (Fig. S5B). The magnitude of chromatin accessibility was strongly correlated with an external ATAC-seq dataset from homogenate PFC tissue (5) (Fig. S5C).

Integration of cell specific OCRs with cell-type-matched ChIP-seq (13) and Hi-C (12) data, identified 56,225 neuron and 49,289 non-neuron enhancer-promoter (E-P) interactions using Activity-By-Contact (ABC) model (14), which linked distal OCRs, covering 17.7–18.6Mb in each cell type (0.60–0.64% of the genome), to 83% of the expressed genes (16,504 of 19,749) (Fig. 1C and see data and materials availability and Fig. S6A). While the majority of distal OCRs were predicted to interact with a single gene, and the frequency of E-P links decreased sharply with distance, about 39% of them were associated with two or more genes (Fig. S6B, Fig. S6D) and only about a quarter were linked to the nearest gene (Fig. S6C).

### Upregulated schizophrenia OCRs in neurons are enriched for schizophrenia risk variants

**Figure 2:**
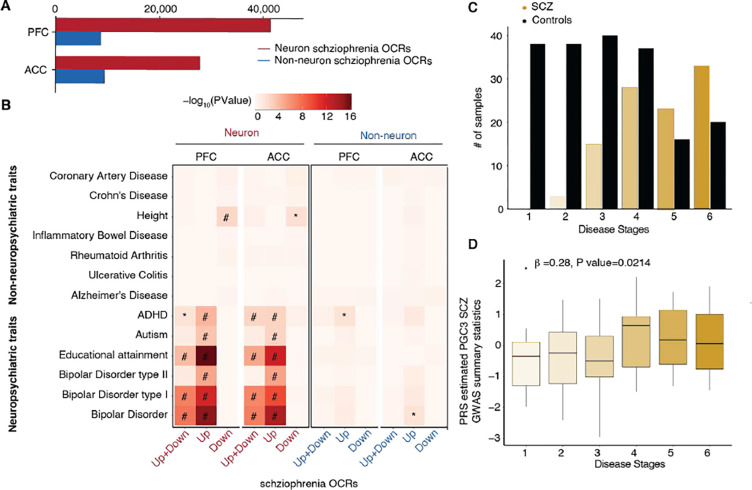
Schizophrenia OCRs and inferred schizophrenia stages in PFC neurons. **A**) Count of schizophrenia OCRs at FDR < .05 using differential analysis of schizophrenia vs controls for PFC and ACC in neurons and non-neurons. **B)** Heatmap of enrichment *P* values of neuropsychiatric and non-neuropsychiatric related GWAS traits. The overlap of OCRs with genetic variants was assessed using LD score regression. ‘#’: significant for enrichment in LD score regression after FDR correction of multiple testing across all tests in the plot (Benjamini–Hochberg test); ‘*’: nominally significant for enrichment. The heritability coefficients of common risk variants that overlap with (1) “Up+Down”: upregulated and downregulated schizophrenia OCRs (41,387(27,771) in PFC(ACC) neurons (red), 8,671(9,405) in PFC(ACC) non-neurons (blue)) (2) “Up”: upregulated schizophrenia OCRs (25,146(15,791) in PFC(ACC) in neurons (red), 3,726(3,075) in PFC(ACC) non-neurons (blue)); and (3) “Down”: dysregulated schizophrenia OCRs (16,241(11,980) in PFC(ACC) neurons (red), 4,945(6,330) in PFC(ACC) non-neurons (blue)). All upregulated (Up) and downregulated (Down) OCRs are with log_2_FC (schizophrenia versus controls) >0 and <0, respectively. **C**) Stratification of neuronal PFC samples by clinical diagnosis as a function of inferred disease stage, where early stage (n=1) to late stage (n=6) is from left to right. **D**) Plot of PRS of neuronal PFC samples calculated using Psychiatric Genomics Consortium (PGC3) schizophrenia GWAS summary statistics, stratified by inferred disease severity stages in neurons from PFC region. Beta estimate and p value are obtained using linear regression model: disease stages ~ PRS + Age + Age^2^ + Sex. Box plots have lower and upper hinges at the 25th and 75th percentiles and whiskers extending to, at most, 1.5xIQR (interquartile range).

We next investigated changes in chromatin accessibility patterns associated with schizophrenia and BD separately for each brain region and cell-type, after correcting for covariates (see methods, Fig. S7–8). Notably, the highest number of schizophrenia-specific alterations (termed “schizophrenia OCRs”) were found in neurons of the PFC (41,387 OCRS, 10.5% of total), followed by the ACC (27,771/7.1% OCRs) (Fig. 2A, Fig. S9A, see data and materials availability). In contrast, non-neurons had significantly lower numbers of schizophrenia-specific alterations in both brain regions. The schizophrenia OCR changes in ACC and PFC were highly concordant (Fig. S9B), indicating comparable disease-associated epigenomic alterations in these regions. For BD, due to less power in the sample size, we found much lower numbers of associations (11–166 OCRs; under 0.1% OCRs) with small effect sizes (Fig. S9A, see data and materials availability).

We then tested whether the observed schizophrenia OCRs were enriched for schizophrenia risk variants. We found magnitude of schizophrenia heritability in neurons was almost two fold for differentially upregulated OCRs in PFC and ACC than all schizophrenia OCRs, whereas downregulated OCRs in neurons had negative and insignificant coefficients in both regions (Fig. S10A, Fig. 2B). The co-localization of schizophrenia risk variants, and other co-heritable traits, such as BD type I, educational attainment and attention deficit hyperactivity disorder (ADHD) with neuron-specific OCRs is in line with the well-established fact that neuropsychiatric traits are enriched in neuronal, rather than non-neuronal, cell populations (16–18) (19) and also exhibited enrichment in upregulated schizophrenia OCRs (Fig. 2B, Table S2).

### Severity of schizophrenia inferred from expression of disease open chromatin regions

Having shown that upregulated schizophrenia OCRs in neurons are enriched with schizophrenia risk variants, we next used the manifold learning method previously employed to investigate Alzheimer’s disease progression in bulk RNA-seq data from postmortem brains (20) to infer disease pseudotime or stages, for each sample. After obtaining the nonlinear function of OCR expression embedded in low-dimensional space, the samples were ordered based on the similarity in expression of upregulated schizophrenia OCRs. Leiden clustering was applied to stratify the samples into six(five) distinct groups in neurons in PFC(ACC) regions denoting an ascending progression of disease severity as shown in Fig. S11A(B) (see methods). This is depicted by control samples being better aligned with less severe disease stages whereas schizophrenia cases were disproportionately represented in groups associated with more severe disease stages in PFC (Fig. 2C) and ACC (Fig. S11C) (see Table S3A–B).

To evaluate the accuracy of the inferred disease stages as a model of disease progression, we calculated a metric called diffusion pseudotime (20, 21) (see methods). We leveraged the pseudotime metric to measure the severity of disease for two groups (schizophrenia and controls) for each brain cortical region. Interestingly, the odds of observing schizophrenia individuals at later pseudotime values from the disease trajectory of neurons is greater than one when compared to controls who have mainly early pseudotime values across both cortical regions (Fig. S11D–E), while no association was found between the inferred disease stages and other demographic and technical measures of samples (Fig. S12).

To measure the link between genetic factors and disease stages, we first estimated schizophrenia PRS for all samples and estimated the correlation with disease status across PFC and ACC region. Figure S11F–G show positive and significant association of PRS with disease status in our study. Next, we hypothesized that per sample inferred disease severity is partially explained by schizophrenia PRS. We observed a significant positive correlation between schizophrenia PRS and disease stages in the PFC (β=0.28, P value = 0.0214, n=140) (Fig. 2D and Table S3C) and ACC ((β=0.27, P value = 0.0172, n=132) using the model disease stages ~ PRS + Age + Age^2^ + Sex (Fig. S11H and Table S3D), providing a convergence of the degree of perturbation in the epigenome of schizophrenia cases with disease heritability.

### Open chromatin regions in *cis*-regulatory landscape colocalize with schizophrenia risk loci

Genomic regulatory elements physically interact at multiple resolutions within the three-dimensional genome for precise gene regulation and overall functioning of cells. Here we focus on identifying physically interacting regulatory elements, named *cis*-regulatory domains (CRDs) which are shown to be delimited by topologically associating domain (TAD) and subTAD boundaries and enriched in binding sites for CCCTC binding factor (CTCF). These domains can be estimated using inter-individual variations in E-P associated histone marks and cell-type specific OCRs (6, 22–24). Using the same analytical framework (see methods, Fig. S13), ~37% of neuronal OCRs were assembled into 6,706 PFC and 6,625 ACC CRDs, and ~33% of non-neuronal OCRs assembled into 4,612 PFC and 4,710 ACC CRDs (Fig. S14A). On average, 21.7(22.1) and 18.9(18.7) OCRs per CRD were present in neuronal and non-neuronal CRDs in PFC(ACC) respectively (Fig. S15A and Table S4). OCRs within CRDs showed cell and region specificity (Fig. S15B). Neuronal and non-neuronal CRDs had a median length of 27.7Kb(26.9Kb) and 42.7Kb(40.3Kb) in PFC(ACC), respectively (Fig. S14B). The identified CRDs were more likely to be located inside cell specific subTADs (Fig. S15C), and CRD borders exhibited strong enrichment for CTCF binding sites (Fig. S15D–H). E-P interactions showed significant enrichment in neuronal and non-neuronal CRDs compared to all OCRs (Fig. S15I). Taken together, cell and region specific CRDs are small structural subunits that constitute E-P interactions which are delimited by subTADs and exhibit characteristics of three dimensional genome organization.

**Figure 3:**
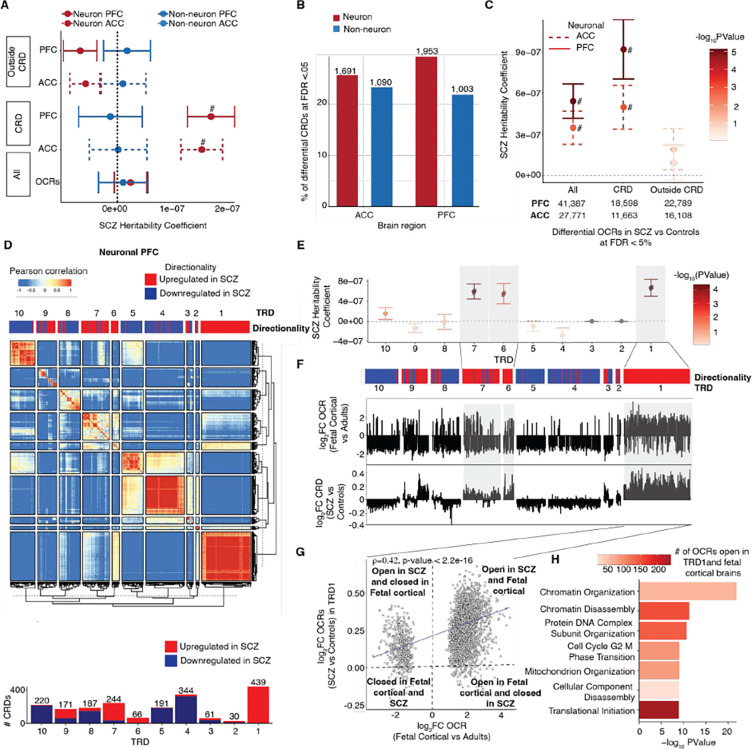
*Cis*-regulatory landscape and trans regulatory domains from hierarchical clustering of neuronal schizophrenia CRDs from the PFC. **A**) Schizophrenia heritability coefficients for neurons (red) and for non-neurons (blue) stratified by (1) “All”: all OCRs (neuronal = 391,420 and non-neuronal =260,431), (2) “CRD”: OCRs inside CRDs (neuronal = 145,533(146,148) and non-neuronal 87,222(87918) in PFC(ACC)) (3) “Outside CRD”: OCRs outside CRDs (neuronal = 245,272(245,887) and non-neuronal 173,209(172,513) in PFC(ACC)). **B**) Bar plot of number of differential CRDs across four datasets identified using a two-stage test at FDR<5%. **C**) Schizophrenia heritability coefficients for neurons (red) and non-neurons (blue) stratified by (1) “All”: all schizophrenia OCRs (2) “CRD”: schizophrenia OCRs inside schizophrenia CRDs and (3) “Outside CRDs”: schizophrenia OCRs outside schizophrenia CRDs. schizophrenia OCRs are identified from the previous section (see Fig. 2) at FDR 5%. **D**) Heatmap depicting hierarchical clustering of trans interactions of 1,953 schizophrenia CRDs across 360 samples from the PFC. The clustering results in ten trans regulatory domains (TRDs) using gamma statistics. The directionality annotation bar plot above the heatmap shows upregulation in red (log_2_SCZ-log_2_Control >0) and downregulation in navy (log_2_SCZ- log_2_Control <0) of schizophrenia neuronal CRDs. Bar plot of Number of up and downregulated schizophrenia neuronal CRDs per TRD from the PFC region. **E**) Coefficients of schizophrenia heritability stratified by TRDs. **F**) Top and bottom bar plots show log_2_FC_fetal_cortical_ (Fetal cortical vs adult controls) and log_2_FC (schizophrenia vs Controls) of neuronal PFC CRD respectively. **G**) Spearman correlation of log_2_FC_fetal_cortical_ compared to log_2_FC (schizophrenia vs Controls) of neuronal PFC CRDs from TRD1 (n=3,056 OCRs). P value is obtained from Spearman’s rank correlation (ρ) test. **H**) Functional pathway enrichment of OCRs from neuronal PFC TRD1 that overlap with fetal cortical specific OCRs. The overlap of OCRs with schizophrenia risk variants A) , C) and E) were assessed using LD score regression. *P* values are from LD score regression. ‘#’: significant for enrichment in LD score regression after Benjamini–Hochberg FDR correction for multiple testing across all tests in the plot (FDR < 5%). Error bars show standard error in schizophrenia heritability from LD score regression.

We examined schizophrenia heritability for OCRs that are inside and outside CRDs. Interestingly, only OCRs inside neuronal CRDs show significant schizophrenia heritability (Fig. 3A and Table S5). Next, we compared the relationship among the disease-associated OCRs, CRDs and “schizophrenia CRDs”. We observed neuronal OCRs with disease signatures to be more likely inside than outside CRDs, based on disease associated t-stats of OCRs (OR =1.331(1.338), *P* < 2.2e-16(2.2e-16) in PFC(ACC)) using logistic regression based generalized linear model (GLM) (see methods). On the other hand, non-neuronal CRDs, showed depletion for disease associated OCRs (GLM results: OR =0.961(0.965), *P* = 1.62e-58(3.07e-45) in PFC(ACC)) (Fig. S16A–B). We then compared the disease-associated OCRs with identified 1,953(1,691) neuronal and 1,003(1,090) non-neuronal “schizophrenia CRDs” that exhibit dysregulation in their mean expression between individuals with schizophrenia and controls in PFC(ACC) (Fig. 3B and methods, see data and materials availability). Genome-wide, approximately 44.9%(41.9%), corresponding to 18,598(11,663) out of 41,387(27,771) neuronal schizophrenia OCRs were found to be encompassed within neuronal PFC(ACC) derived schizophrenia associated CRDs. Similarly, approximately 30.6%(27.3%) of the non-neuronal schizophrenia OCRs, amounting to 2,656(2,569) out of 8,671(9,405), were identified as being contained within non-neuronal schizophrenia CRDs. Across all four datasets, schizophrenia OCRs tend to be clustered inside schizophrenia CRDs rather than outside schizophrenia CRDs (Fig. S16C). Within the neuronal schizophrenia CRDs, a higher proportion of schizophrenia OCRs is linked to disease associated transcripts (Fig. S16D–E). Schizophrenia OCRs in neuronal schizophrenia CRDs had over 1.5-fold increase in schizophrenia heritability compared to all schizophrenia OCRs across the PFC(ACC) regions (Fig. 3C), while schizophrenia OCRs outside neuronal schizophrenia CRDs had no significant schizophrenia heritability (Table S6). These findings suggest chromatin interactions from 37% of OCRs that comprise neuronal CRDs delimit the OCRs that are significantly enriched for schizophrenia risk loci from the rest of the genome, and that these can be explored to identify disease associated expression changes.

### Trans interactions of CRDs in schizophrenia brains are enriched for neurodevelopmental OCRs

We next examined whether the interaction between disease associated CRDs could identify trans-regulatory domains (TRDs) that act as hubs for convergence among the degree of perturbation in the epigenome of schizophrenia cases with disease heritability. The analytical workflow from CRD to TRDs is shown in schematic displayed in Fig. S17A. We applied hierarchical clustering on a correlation matrix of expression of schizophrenia CRDs across all neuronal samples and Gamma statistics (25) to identify a total of 10 neuronal TRDs (see methods and Fig. S17B). While most TRDs exhibit a mix of upregulated and downregulated CRDs, there is one particular TRD (TRD1) that stands out with a predominant upregulation of CRDs in PFC and ACC (Fig. 3D and Fig. S18A; see data and materials availability). TRD1 also had the highest proportion of schizophrenia OCRs compared to other TRDs (Fig. S17C). We then examined the association of TRDs with schizophrenia risk loci. Three TRDs (1, 6, 7) out of ten in PFC (Fig. 3E and see data and materials availability) and two TRDs (1, 2) out of ten in ACC (Fig. S18B and see data and materials availability) had significant and positive coefficients of schizophrenia heritability for OCRs contained within them. One of the common characteristics of TRDs (1, 6, 7) in PFC and TRDs (1,2) in ACC, referred to as “schizophrenia TRDs” hereon, was a higher proportion of upregulated schizophrenia OCRs out of the total number of schizophrenia OCRs contained within them (Fig. S17D). To connect these OCRs with molecular mechanisms, we performed functional pathway analysis of OCRs in “schizophrenia TRDs”. Across PFC and ACC, TRD1 shows enrichment in biological processes such as membrane organization, cellular component organization, synaptic maturation and regulation of synaptic plasticity (see data and materials availability). PFC TRDs 6 and 7, and ACC TRD2 showed enrichment for the neuronal system, Rho GTPases and signalling proteins. In summary, we noticed multiple neurodevelopmental signatures such as chromatin organization and synaptic maturation in TRDs, however, it wasn’t clear which specific molecular mechanism was present in a specific TRD.

Given the enrichment of schizophrenia TRDs with neurodevelopmental signatures such as chromatin organization and synaptic maturation, we hypothesized that schizophrenia TRDs contain OCRs that are active during neurodevelopment. To explore this hypothesis, we first performed ATAC-seq in fetal cortical plate (post-conception weeks: 19–24; n=4 samples) (see methods) and compared it with adult controls from PFC neurons (aged >30 years, n=6) (29) to identify OCRs specific to early brain development. Upon comparison with fetal cortical samples, the upregulation in schizophrenia associated changes in CRDs within TRDs was strongly correlated with upregulation in early brain development OCRs in the PFC (Fig. 3F) and ACC (Fig. S18C). Thus, chromatin regions that are open in fetal cortical samples, are more likely to also be open in adult schizophrenia samples than in adult controls. Comparison of development-specific OCRs with schizophrenia associated changes in OCRs showed a significant correlation in TRD1 (ρ=0.42 at p value < 2.2e-16, n=3,056 OCRs) in PFC (Fig. 3G) and (ρ=0.46 at p value < 2.2e-16, n=3,149 OCRs) in ACC (Fig. S18D). In the PFC, the correlation of TRD6 with developmental-specific OCRs was slightly weaker (ρ=0.24, p value=3.47e-06, n=371 OCRs). However, no significant correlation was observed in PFC-TRD7 (ρ=0.02, p value=0.587, n=523 OCRs) and ACC-TRD2 (ρ=0.11, p value=.04, n=371 OCRs). Functional pathway analysis in OCRs that are upregulated in fetal cortical samples and PFC-TRD1, ACC-TRD1 and PFC-TRD6 identified biological processes related to cell adhesion, translation initiation, chromatin organization and chromatin disassembly (Fig. 3H, Fig. S18E, and see data and materials availability).

### Schizophrenia-specific neurodevelopmental TRDs are associated with early and late stages of brain development

**Figure 4:**
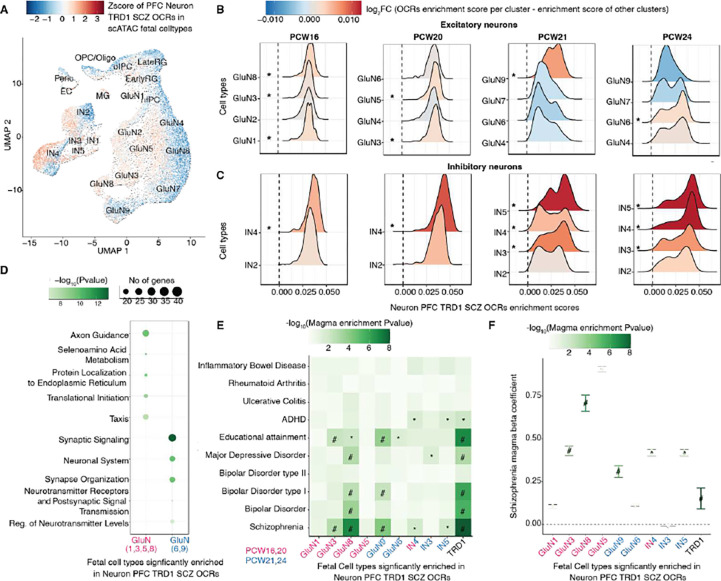
Analysis of cell-type specificity of neuronal PFC schizophrenia OCRs in fetal cortical scATAC-seq data. **A**) Uniform Manifold Approximation Projection (UMAP) plot of fetal cortical scATAC-seq data from Trevino *et al.* in which each cell type is colored by Z-scores of schizophrenia OCRs in neuronal PFC TRD1. The cell types include early radial glia (EarlyRG), late radial glia (LateRG), oligodendrocyte progenitor cell/oligodendrocyte (OPC/Oligo), neuronal intermediate progenitor cell (nIPC), oligo intermediate progenitor cell (oIPC), glutamatergic neuron (GluN), interneuron (IN), endothelial cell (EC), microglia (MG), and pericytes (Peric). Distribution of enrichment scores stratified by two major classes of cell types: **B**) excitatory neurons (nine subtypes of glutamatergic neurons) and **C**) inhibitory neurons (five subtypes of inhibitory neurons). The color represents the magnitude of coefficient of association of enrichment scores of schizophrenia OCRs from neuronal PFC-TRD1 at each developmental stage separately for each cell types, using the model (enrichment score ~ celltype + (1|sample ID) where cell types are grouped as the cell type of interest vs. all other cell types. * depicts the cell types that are significantly enriched from the latter test and also have higher enrichment than the schizophrenia OCRs outside TRDs. **D**) Functional pathway analysis conducted on the early and late fetal glutamatergic specific marker genes that are also annotated by schizophrenia OCRs in TRD1. **E**) Heat map of MAGMA enrichment *P* values of brain-related and non-brain related GWAS traits and **F**) Coefficients of enrichment of common schizophrenia risk variants using MAGMA in fetal cell specific marker genes that are also annotated to schizophrenia OCRs within neuronal PFC TRD1. The x-axes in **E**) and **F**) are 1) early fetal (pcw16+20) glutamatergic (GluN 1,3,5,8) (magenta), 2) late fetal (pcw21+24) glutamatergic (GluN 6,9) (blue), 3) early+late fetal (pcw16+20+21+24) inhibitory (IN 4) (magenta+blue), 4) late fetal (pcw21+24) inhibitory (IN 3,5) fetal cell types (blue) and all ABC mapped schizophrenia OCRs to genes within neuronal PFC TRD1 (black). ‘#’: significant MAGMA enrichment coefficient after FDR correction of multiple testing across all tests in the plot (Benjamini–Hochberg test); ‘*’: nominally significant for enrichment in **E**) and **F**).

After observing the neurodevelopmental signatures in neuronal schizophrenia TRDs, we aimed to identify specific neuronal cell types and brain developmental stages where these disease-associated perturbations occur. To accomplish this, we tested whether OCRs in neuronal TRDs are expressed in fetal cell types using an atlas of single-nucleus ATAC-seq data generated from human fetal cortical samples spanning 8 weeks across mid-gestation: specifically, at pcw16, 20, 21, and 24 ( see methods) (26). The early stages (pcw16 and 20) are characterized by cell division and neural precursor proliferation, whereas the later stages (pcw21 and 24) are associated with cell migration and maturation. Firstly, the schizophrenia OCRs in fetal cell types displayed positive z-scores for TRD1 in both PFC and ACC (Fig. 4A and Fig. S19A), compared to other schizophrenia TRDs (Fig. S20). Secondly, TRD1 consistently showed positive and significant differential enrichment scores in GluN(1,3,5,8) across PFC and ACC during early fetal stages of brain development (pcw16+20). In contrast, GluN(6,9) showed significant association at later stages of development (pcw21+24) (Fig. 4B and Fig. S19B). Regarding inhibitory neurons, IN(3,4,5) displayed positive and significant differential enrichment scores in PFC and ACC at all stages of brain development (Fig. 4C and Fig. S19C). None of the other schizophrenia TRDs showed association with fetal cell types (Table S7A–B).

Next, we explored the specific molecular mechanism underlying these developmental stage specific glutamatergic, and inhibitory neurons, by intersecting cell types marker genes with E-P linked genes within TRD1 (see methods). Schizophrenia OCRs within TRD1 mapped to early stage genes from GluN(1,3,5,8) were enriched for DNA transcription and cell cycle processes (Fig. 4D and Fig. S19D); the late stages genes from GluN(6,9) were associated with synapse organization and maturation. On the other hand, schizophrenia OCRs within neuronal TRD1 mapped to early and late stage inhibitory neuronal genes were less in number resulting in underpowered pathway analysis that failed to pass multiple testing correction (Table S7I–J). Lastly, we asked whether these developmental cell specific genes have significant enrichment for schizophrenia risk loci. Remarkably, Glu (3,8,9) and IN(4,5) showed significant and positive coefficient of enrichment for schizophrenia risk loci and other co-heritable neurodevelopmental traits, including BD, ADHD, and educational attainment. No significant enrichment was observed for non-brain related traits, such as inflammatory bowel disease and rheumatoid arthritis. Taken together, these findings show that TRD1 isolates a set of OCRs that are upregulated in adult schizophrenia brains, are specific to neurodevelopmental glutamatergic and inhibitory neurons, and correspond to disrupted DNA binding related cellular processes during early stages and misregulated synaptic maturation during late stages of brain development.

### Neurodevelopmental TRDs stratify the schizophrenia cases and controls using polygenic risk scores

**Figure 5:**
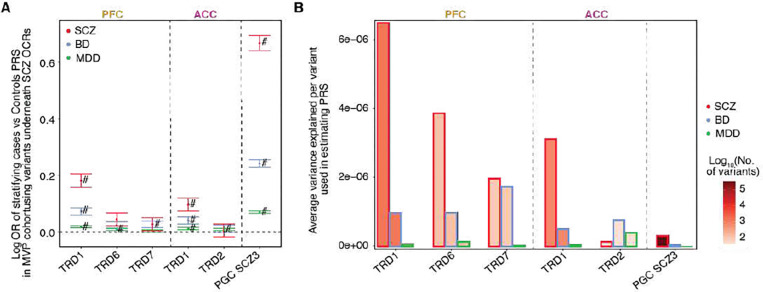
Trans Regulatory Domain one (TRD1) in PFC and ACC can stratify schizophrenia cases and controls in MVP cohort. For schizophrenia TRDs: TRD1, TRD6 and TRD7 from PFC region and TRD1, TRD2 from ACC region. **A**) Plot of logOR from logistic regression of PRS of 203,078 individuals from MVP cohort estimated using the SNPs in OCRs from these TRDs to predict their case-control status. Error bars show standard error in estimating OR from logistic regression. **B**) Barplot of the average variance explained per variant in estimating PRS of SCZ, BD and MDD individuals (bar contour color is red, blue and green respectively). Bar fill color corresponds to the number of variants considered in each group for PRS estimation (PFC TRDs: 1,388 for TRD1, 139 for TRD6 and 103 for TRD7; ACC TRDs: 815 for TRD1 and 46 for TRD2; PGC3 SCZ: 350,882).

Finally, we explored whether the cumulative effect of genetic variation underlying schizophrenia TRD predicts an individual’s genetic susceptibility to schizophrenia and co-heritable traits, including BD and MDD in an independent cohort from the Million Veterans Program (MVP), which includes 1,957 (7,177; 120,067) schizophrenia (BD; MD) non-overlapping cases and 194,019 controls. Remarkably, PRS scores constructed with variants within schizophrenia OCRs within TRD1 (subsetted from the full PGC3 SCZ GWAS summary statistics) showed the highest odds of association with SCZ diagnosis when compared to other schizophrenia TRDs in the MVP cohort (Fig. 5A–B). After accounting for the varying number of genetic variants within each TRD (see methods), TRD1 showed the highest average variance explained per variant compared to other schizophrenia TRDs across both cortical regions and variants across the whole genome (Fig. S21A). We replicated this main finding in the CommonMind cohort (312 SCZ cases and 320 controls), where the variants withins OCRs in TRD1 showed the highest odds of classifying cases and controls and the highest average variance explained per variant in estimating PRS when compared to other schizophrenia TRDs (Fig. S21B–C). We observed a similarly strong correlation between PRS and stages in the PFC (Spearman rho=0.17, P value = 0.044, n=140) (Fig. S21D) and ACC (Spearman rho=0.21, P value = 0.013, n=132) (Fig. S21E), further pointing to the convergence of disease severity based on epigenetic dysregulation with genetic liability for schizophrenia.

## Discussion

We have created a large and comprehensive resource of 1,393 chromatin accessibility samples derived from neurons and non-neurons in the PFC and ACC of human brain specimens, including controls and cases with schizophrenia and BD. Differential chromatin accessible regions in neurons of schizophrenia brains were significantly associated with schizophrenia risk loci when compared to controls, which is consistent with a previous observation in the same cohort that perturbations in the enhancer-promoter-associated epigenetic marker, H3K27ac, are enriched in schizophrenia risk loci (6). Overall, these studies point to convergence among cell type-specific alterations in non-coding regions of the genome with the underlying genetic risk architecture of schizophrenia. We note the absence of results in BD due to less power in the sample size resulting in much lower numbers of associations with small effect sizes.

The concept of severity stages is well-established in diseases, such as AD (27), but has not been extensively explored in psychiatric disorders due to the absence of disease related molecular and pathological markers. A recent study in AD, applied the manifold learning method on bulk RNA-seq data from postmortem brains and identified severity stages that match disease stages based on pathological criteria (20). Using a similar approach, we derived a “disease pseudotime score”, stratifying samples into distinct clusters based on the extent of perturbation in the expression of disease-associated OCRs. The inferred order of samples was concordant with schizophrenia PRS across both cortical regions examined. This finding has the potential to open new avenues for the development of targeted drugs specific to different stages of the disease, similar to the concept of exploiting PRS for precision medicine in other disease fields (28–30).

In the next part of our study, we identified *cis*-regulatory domains (CRDs) of physically interacting chromatin regions, that constitute over 33% of genome-wide OCRs that are enriched for schizophrenia risk loci to CRDs only in neurons. To explore disease-relevant molecular mechanisms confined to higher order chromatin structures, we performed hierarchical clustering on disease associated CRDs based on their inter-individual correlation to identify trans regulatory domains (TRDs). Notably, one TRD (termed TRD1), which exhibited a high abundance of upregulated OCRs across both cortical regions, displayed a strong correlation with OCRs upregulated in fetal cortical samples compared to adult controls. By calculating the expression scores of schizophrenia OCRs from TRD1 in scATAC-seq data from fetal samples, we were able to pinpoint critical cell types in glutamatergic neurons at both early and late stages of development. The schizophrenia OCRs mapped to early stage glutamatergic neurons were related to DNA binding and translation, while schizophrenia OCRs mapped to late stage glutamatergic neurons showed processes related to synapse maturation and organization. These findings are concordant with the theory that non-coding regions, which host the majority of common schizophrenia risk variants, are linked to neurodevelopment.

Our findings lay the groundwork for characterizing neurodevelopmental cell types within diseased chromatin regions in adult postmortem brains that experienced disruptions during brain development. By gaining a deeper understanding of the roles played by neurodevelopmental cell types, we can establish connections between the molecular mechanisms of the disease and its manifestation during brain development, ultimately linking them to the symptoms observed in adulthood. Furthermore, there is compelling evidence supporting a neurodevelopmental basis for schizophrenia (31–33). Thus, this analysis provides a crucial link in interpreting the impact of risk mutations during the critical period of brain development, unravelling the susceptibility to schizophrenia. It should be noted that future improvements in the analysis could involve generating and analyzing scATAC-seq data from postmortem brains affected by schizophrenia and controls. This approach would allow for a more accurate mapping of disease-affected cell types to neurodevelopmental cell types, further enhancing our understanding of the condition.

Lastly, from this work, we provide unique resources of (1) dysregulated cell and region-specific chromatin regions in a cohort of schizophrenia and BD and (2) genome-wide CRDs that encompasses physically interacting OCRs and confines the schizophrenia risk loci from the rest of the OCRs. We also demonstrate (3) the stratification of schizophrenia samples based on the degree of perturbed epigenome. We (4) introduced a unique workflow based on hierarchical clustering of correlation of differential CRDs to map these neurodevelopmental OCRs to critical cell types using scATAC-seq data and (5) obtained a list of epigenomic marker OCRs that covers only 0.25–0.39% of risk SNPs and can stratify the cases and controls. The analyses and findings in this study provide the foundation for future investigations towards quantifying the contribution of specific cell types that are affected during brain development and later in life in brains of affected individuals. By understanding the extent of the contribution of specific cell types, we can gain valuable insights into the underlying mechanisms of the disease and potentially develop quantitative measures for assessing disease severity at a specific age, paving the way for personalized approaches to diagnosis and treatment in individual patients.

## Supplementary Material

Supplement 1

## Footnotes

**Additional information:** Supplementary information is available for this paper at https://www.synapse.org/#!Synapse:syn52264219. Reprints and permissions information is available at www.nature.com/reprints. Correspondence and requests for materials should be addressed to P.R.
