# Supplementary material for: The neuronal chromatin landscape in adult schizophrenia brains is linked to early fetal development
Source: Res Sq. 2023 Oct 2:rs.3.rs-3393581. Preprint. [Version 1] doi: 10.21203/rs.3.rs-3393581/v1 (PMC10602154; doi:10.21203/rs.3.rs-3393581/v1)
Supplement: Supplement 1 [file NIHPPRS3393581V1-supplement-1.pdf]

Supplementary Information

The neuronal cis-regulatory chromatin landscape in adult schizophrenia brains is linked to early brain development.

This PDF file includes:

- Materials and methods
- Captions for Supplementary Figures S1-S21
- Supplementary Figs. 1-21
- Captions for Supplementary Tables S1-S13

Other Supplementary Materials/ Data Files for this manuscript include the following:

- Supplementary Tables 1-13 (.xlsx)

Materials and Methods

Description of the post-mortem brain samples

Frozen brain tissue derived from ACC (anterior cingulate cortex/Broadmann area 10) and PFC (prefrontal cortex/Broadmann area 9 and 46) were obtained from three separate brain banks, described below. This cohort of schizophrenia (SCZ), bipolar or other affective/mood disorder (AFF) cases and control subjects was assembled after applying stringent inclusion/exclusion criteria. All involved subjects had to meet the appropriate diagnostic DSM-IV criteria, as determined in consensus conferences after review of medical records, direct clinical assessments, and interviews of family members or care providers. All tissue donors were from the Icahn School of Medicine at Mount Sinai (MSSM), University of Pittsburgh (PITT) brain banks and NIMH Human Brain Collection Core (HBCC). **Table S1A-D** tabulates the demographic information at sample level of the present study, including sex, age of death, and ethnicity, stratified by cell type, brain region, institution, and diagnosis. The link to the complete demographic and clinical information of the present study population is provided on synapse platform (syn52264219).

Generation of ATAC-seq libraries and sequencing

50mg of frozen brain tissue was homogenized in chilled lysis buffer (0.32M Sucrose, 5 mM $CaCl_2$, 3 mM Magnesium acetate, 0.1 mM, EDTA, 10mM Tris-HCl, pH8, 1 mM DTT, 0.1% Triton X-100) and filtered through a 40μm cell strainer. Filtered lysate was underlaid with sucrose solution (1.8 M Sucrose, 3 mM Magnesium acetate, 1 mM DTT, 10 mM Tris-HCl, pH8) and subjected to ultracentrifugation at 24,000 rpm for 1 hour at 4°C. Pellets were re-suspended in 500μl DPBS supplemented with 0.1% BSA. anti-NeuN antibody (1:1000, Alexa488 conjugated, Millipore Cat #MAB377X) was added and samples incubated, in the dark, for 1hr at 4˚C. Prior to FANS sorting, DAPI (Thermoscientific) was added to a final concentration of 1μg/ml. DAPI positive neuronal (NeuN+) and non-neuronal (NeuN-) nuclei were isolated using a FACSAria flow cytometer (BD Biosciences).
ATAC-seq libraries were generated using an established protocol (*1*). Briefly, 50,000 or 75,000 sorted nuclei were pelleted at 500 g for 10 min at 4°C. Pellets were re-suspended in transposase reaction mix (22.5 μL Nuclease Free H_2O, 25 μL 2x TD Buffer; Illumina Cat #FC-121-1030) and 2.5 μL Tn5 Transposase (Illumina Cat #FC-121-1030) on ice and the reactions incubated at 37°C for 30 min. Following incubation, samples were purified using the MinElute Reaction Cleanup kit (Qiagen Cat #28204, and libraries generated using the Nextera index kit; Illumina Cat #FC-121-1011) as previously described (*3*). Following amplification, libraries were resolved on 2% agarose gels and fragments ranging in size from 100-1000bp were excised and purified (Qiagen Minelute Gel Extraction Kit – Qiagen Cat#28604). Next, libraries were quantified by quantitative PCR (KAPA Biosystems Cat#KK4873) and library fragment sizes estimated using Tapestation D5000 ScreenTapes (Agilent technologies Cat# 5067-5588). ATAC-seq libraries were sequenced by Hi-Seq 2500 and Novaseq 6000 (Illumina) obtaining 50bp paired-end reads.

Processing of data

We have implemented our in-house pipeline for processing of ATAC-seq data previously used for other studies (*2*, *3*). The detailed steps are explained below.

Alignment of raw sequencing files

The raw reads were trimmed with Trimmomatic (*4*) and then mapped to human reference genome GRCh38 analysis set reference genome with the pseudoautosomal region masked on chromosome Y with the STAR aligner (v.2.7.0e) (*5*). To correct for allelic bias resulting from person-specific genome variation, we ran STAR with enabled WASP module (*6*) as we provided both ATAC-seq FASTQ file and WGS file or SNP array genotype of corresponding individual. This yielded for each sample a BAM

file of mapped paired-end reads sorted by genomic coordinates. From these files, reads that mapped to multiple loci or to the mitochondrial genome were removed using samtools (*7*) and duplicated reads were removed with PICARD (v2.2.4; http://broadinstitute.github.io/picard). Quality control metrics were reported with phantompeakqualtools (*8*), ataqv (*9*), and Picard. All ATAC-seq QC metrics are summarized in **Table S1F** and details at sample level are provided on synapse platform (syn52264219).

Genotype calling
For ATAC-seq, genotypes were called using GATK (v3.5.0) (*10*). In brief, these steps were performed: (1) indel-realignment; (2) base score recalibration; and (3) joint genotype calling across all samples for variants with a phred-scaled confidence threshold >= 10. All clustered variants, variants in ENCODE blacklisted regions of the genome (*11*), and variants not in dbSNP v151 (*12*) were not considered. Read depth was not used for filtering. Finally, only variants with minor allele frequencies (MAF) ≥ 25% were considered. Pairwise genotype concordance was carried out amongst all ATAC-seq samples and whole genome sequencing (WGS) (or SNP-array samples for donors without WGS) using the kinship coefficient from KING v1.9 (*13*) and considering only the variants called in the ATAC-seq data. Cut-offs for the kinship values were selected based on the samples that were expected to be from the same brain versus those that were not. Using these cut-offs, we identified and resolved all sample swaps. We also reported potentially contaminated libraries that were matching multiple genotypes (or no genotype), mostly due to mistakenly used identical barcodes within the same pools for more than one sample. Those libraries were removed from the dataset.

Peak/OCR Calling
Peaks or OCRs calling was done as previously described (*2*, *3*) with a modification of FDR threshold to 0.01.

Metrics used for quality control
For each sample, the following quality control metrics were used: the total number of initial reads; the number of uniquely mapped reads; the fraction of reads that were uniquely mapped; further metrics from the STAR aligner; the GC content, duplication and insert metrics from Picard; the rate of reads mapping to the mitochondrial genome; the PCR bottleneck coefficient (PBC), which is an approximate measure of library complexity estimated as uniquely mapped non-redundant reads divided by the number uniquely mapped reads; TSS enrichment in housekeeping genes (calculated per ENCODE ATAC-seq data standards); the normalized strand cross-correlation coefficient (NSC) and the relative strand cross-correlation coefficient (RSC), which are metrics that use cross-correlation of stranded read density profiles to assess sample quality independently of peak calling; and finally the fraction of reads in peaks (FRiP), which is the fraction of reads that fall in detected peaks, the fraction of reads in only blacklisted peaks (*11*), and the ratio between these two metrics (to calculate these metrics, the consensus set of peaks was used). The main quality metrics are shown in **Table S1F** and details at sample level are provided on the synapse platform (syn52264219).

Annotation of OCRs to genomic elements
Neuronal and non-neuronal OCRs were annotated to TSS, exon, 5′ UTR, 3′ UTR, intronic or intergenic regions using ChIPSeeker (version 1.18.0). All OCRs within ±3 kb distance from TSS of a gene were annotate as promoters. **Fig. S5A** shows the distribution of neuronal and non non-neuronal OCRs annotated to (1) promoters, (2) introns, (3) distal intergenic and (4) exon and UTRs using the "TxDb.Hsapiens.UCSC.hg38.knownGene" transcript database in-built in the ChIPSeeker package.

Annotating OCRs to genes using ABC model

We used Activity-by-contact model (ABC, v.0.2) (*14*) to construct a comprehensive regulatory map of enhancer-promoter (E-P) interactions in neuronal and non-neuronal cell types of the two investigated brain regions (DLPFC and ACC). This model requires: (1) contact frequency between putative enhancers and promoters of regulated genes; and (2) enhancer activity data. Contact frequency matrices were generated from neuronal and non-neuronal Hi-C datasets composed of eight post-mortem human brains (*2*). Here different neocortical regions (dorsolateral prefrontal cortex, orbital frontal cortex, and anterior prefrontal cortex) were profiled across multiple donors aged 34-103 years. Enhancer activity data was represented by the cell type and brain region specific ATAC-seq signal (current study) and the H3K27ac ChIP-seq signal. ChIP-seq data was generated in a subset of ten controls (STG and EC; age of donors ranged between 61-103 years) (*15*). In accordance with the authors' directions, we filtered out predictions for genes on chromosome Y and lowly expressed genes (genes that did not meet inclusion criteria in our RNA-seq dataset). We used the default threshold of ABC score (a minimum score of 0.02), the default screening window (5MB around the TSS of each gene), and we skip ABC calculation for ubiquitously expressed genes (using default ABC list of 806 genes).

Analysis of differentially accessible OCRs (schizophrenia OCRs)

To assess which OCRs showed differential accessibility in SCZ- and BD-related phenotypes, we analyzed the accessibility statistically. For this, chromatin accessibility was estimated by the number of ATAC-seq reads overlapping a given OCR. The more overlaps seen with an OCR the more accessible the more accessible the OCR was considered. The statistical analysis encompassed the following steps.

Read count and OCR filtering

The starting point here were the sample by OCR matrices (separately for neuronal and non-neuronal samples) of read counts generated as described in the preceding section. From these matrices, we excluded OCRs that were lowly accessible by only keeping OCRs that had at least 1 count per million reads in at least 10% of the samples. This removed 742 and 4 neuronal and non-neuronal OCRs, respectively, and resulted in a final read count matrices of 690 neuronal samples by 391,420 OCRs and 703 non-neuronal samples by 260,431 OCRs. Next, the read counts were normalized using the trimmed mean of M-values (TMM) method (*16*).

Exploration of covariates and model selection

To explore the effect of technical and biological covariates, we first did a principal component analysis (PCA) on the normalized read counts to identify high-variance components explaining more than 1% of the variance. We then accessed the correlation of covariates with the PCs and selected those that showed a significant correlation with one or more PCs at a lenient FDR cut-off of 0.2 as candidate covariates for the analysis of differential chromatin accessibility. This encompassed 64 covariates including FRiP, GC content metrics, mapping metrics, insert metrics, the fraction of reads mapping to the mitochondrial genome, PBC, RSC, and barcode. These covariates were subsequently assessed as detailed in the following.

Next, the starting point for modeling the chromatin accessibility was chosen as the variables "brain region by diagnosis status" (2x2=4 levels) and "sex" (2 levels) for a base model. The variable "sex" was included as it is known to have a strong effect on a few OCRs primarily located on the gonosomes. To assess which covariates should be included in order to have a good average model of OCR accessibility we employed the Bayesian information criterion (BIC). In particular, it was for each additional covariate tested how many OCRs showed an improved BIC score minus how many showed

a worse BIC score when the covariate was included in the linear regression model compared to when it wasn't. Here, a covariate was required to improve mean BIC per OCR by at least 10 in at least 2% of OCRs in order for it to be included in the final model. If there are some covariates fulfilling this criteria, the best performing of them is added into the base model and the remaining covariates are tested again. For neuronal dataset, the following numerical covariates were selected: "GC 80-100%" (i.e., normalized coverage over each quintile of GC content ranging from 80 – 100%), "Fraction of reads in peaks (FRiP)", "Fraction of unmapped reads", "GC 20-39%", "AT dropout", "GC 40-59%", and "Age at the time of death". For non-neuronal dataset, the following numerical covariates were selected: "GC 20-39%", "GC 80-100%", "GC 0-19%", "Age at the time of death", "Fraction of reads in blacklisted peaks (FRiBP)".
Subsequently 14 categorical covariates were considered for inclusion due to the higher number of degrees of freedom of each covariate. None of these fulfilled the BIC criteria for inclusion. Finally it was considered if the selected numeric covariates affected chromatin accessibility as a quadric term by testing the squared FRiP for inclusion. It did not meet the BIC inclusion criteria and was therefore not added to the model. Resultantly the final neuronal and non-neuronal models jointly encompass 14 and 12 degrees of freedom, respectively (**Fig. S7**).

Statistical analysis of differences in chromatin accessibility
The normalized read count matrices from voomWithDreamWeights (variancePartition package (*17*, *18*)) were then modeled for each brain region (i.e.,PFC, ACC), and diagnosis (i.e., SCZ, BD, and Controls) by fitting weighted least-squares linear regression models estimating the effect of the right hand side variables on the expression/accessibility of each feature:

- neuronal OCR expression ~ brain region:diagnosis + sex + GC_80-100% + FRiP + Fraction_unmapped_reads + GC_20-39% + AT_dropout + GC_40-59% + Age_of_death
- non-neuronal OCR expression ~ brain region:diagnosis + sex + GC_20_39% + GC_80-100% + GC_0-19% + Age_of_death + FRiBP

Since our dataset contains three samples per individual, we ran differential analysis by *dream* method (variancePartition package (*17*, *18*)). *Dream* method properly model correlation structure and, thus, keep false discovery rate lower than the other commonly used methods for this purpose.

Analysis of differentially expressed genes

The analysis of differential gene expression follows the same protocol as previously described analysis of chromatin accessibility. Therefore, a description will focus on explanation of changes of parameters in the pipeline.

Read count and OCR filtering
The initial read count matrix of the CMC RNA-seq dataset (*19*) consisted of 58,930 genes quantified for 1,818 samples (only samples from DLPFC and ACC brain regions with SCZ, BD, and Control diagnosis status were selected). Then, we calculated the correlation of each sample to all other samples and removed the samples with markedly different correlation, i.e., the difference of mean correlation of the given sample with the rest of the dataset and mean correlation of all pairs of samples within the dataset was more than four times higher than the standard deviation calculated upon all pair's correlation. We only kept protein-coding genes with gene expression of at least 1 count per million reads in at least 30% of the samples. These filters led to the exclusion of 66 samples and 44,024 genes so the resultant read count matrix consisted of 1,818 samples by 14,906 genes. We performed quantile

normalization of the data followed by normalization using the trimmed mean of M-values (TMM) method (*16*).

Exploration of covariates and model selection

The following covariates were selected by BIC method to be added to the base covariates, i.e. "region by diagnosis status" (2x3=6 levels), "sex" (2 levels), and "institution" (4 levels), "Expression profiling efficiency" (numeric, squared), "Intragenic rate" (numeric, squared), "GC 80-100%" (numeric), "AT dropout" (numeric), "age at the time of death" (numeric), "GC 20-39%" (numeric), "intronic rate" (numeric), "GC 60-79%" (numeric), intergenic rate (numeric, squared), "duplication rate" (numeric). We required that at least 5% of the peaks showed a change of 2 in the BIC score, corresponding to "positive" evidence against the null hypothesis (*20*). The relatively high number of covariates can be explained by the study design which employed homogenate tissues, not FANS-sorted nuclei. Therefore, the model needed to cope with naturally different neuronal / non-neuronal composition in different brain regions. The final model jointly encompassed 24 degrees of freedom.

Identification of SCZ stages using manifold learning

To identify SCZ stages, neuronal and non-neuronal schizophrenia OCRs were first filtered based on FDR corrected p-values with thresholds of FDR < .0001 in PFC (n=2,411 neurons upregulated schizophrenia OCRs), FDR < .001 in ACC (n=1,495 neurons upregulated schizophrenia OCRs), FDR < .0001 in PFC (n= non-neurons upregulated schizophrenia OCRs), and FDR < .001 in ACC (n= non-neurons upregulated schizophrenia OCRs). Filtered expression matrices of cell-region specific OCRs were then projected onto the first 50 principle components and a nearest neighbour graph was constructed using the UMAP neighbour kernel (*21*). Diffusion maps (*22*) were calculated with the resulting neighbour graph using the kernel normalisation introduced in (*23*) (see **Fig. S11A-B**). We then constructed another UMAP neighbour graph using the first 15 diffusion coordinates and performed Leiden clustering (*24*) (resolution parameter = 1) to identify SCZ stages in diffusion map space. A total of 6(5) leiden clusters were obtained from PFC(ACC) regions in neuronal samples (see **Fig. 2C** and **S11C**). A diffusion pseudotime calculation was then performed according to (*23*) with a root node in the cluster with the highest density of control samples. All analysis was performed in scanpy using default parameters (see **Fig. S11D-E**).We limited the pseudotime estimation to neuron PFC and ACC samples only because of noisy diffusion maps for non-neurons PFC and ACC OCRs expression matrices.

The diffusion maps of the non-neuronal OCRs were notably noisier than those of the neuronal OCRs, leading to worse clustering and SCZ staging. One possible reason for this is the difference in spectral gaps, i.e. the difference between the eigenvalues associated with the first and second principal components of the data. When the data is Gaussian distributed, large spectral gaps lead to clean diffusion maps which follow the shapes shown in (see **Fig. S11A-B**), while smaller gaps lead to noisier ones. One biological reason for this is that the non-neuronal samples contain a mixture of cell types (astrocytes, oligodendrocytes, and microglia) as opposed to the neurons which only contain different subtypes. The first and second principal components of the mixture distribution can, in general, be orthogonal to the first and second principal components of each individual cell distribution and in such a case the spectral gap of the mixture distribution can be decreased and, in some cases, disappear entirely. If one cell type dominates the mixture distribution with a large number of samples and large variance, its principal components will dominate the mixture distribution while the other cell types will shrink the overall spectral gap of the mixture relative to the dominant cell type. This leads to decent diffusion maps for the first couple of diffusion components, followed by noisy diffusion components that deviate from the expected structure.

Polygenic Risk Scores

Million Veterans Program cohort

The Million Veteran Program (MVP) is a mega-biobank that has been previously described (*25–27*). On top of quality control performed by the MVP Core Team, we perform additional quality control (sex check, relatedness, minor allele frequency, hardy weinberg equilibrium, missingness, etc..) recommended for similar studies (*28*) using electronic medical data v20.1. For this study, we only utilise the European (EUR) population as defined by HARE(*29*) using the 1000 Genomes Project reference (*30*).

PRS estimation

Genotyping specifications for the CMC SNP array data used in the PRS analyses have been described previously(*19*, *31*). For the dataset used in these analyses specifically, marker positions were lifted-over to GRCh38. Pre-imputation processing then included running the quality control script HRC-1000G-check-bim.pl from the McCarthy Lab Group (https://www.well.ox.ac.uk/~wrayner/tools/), using the Trans-Omics for Precision Medicine (TOPMed) reference(*32*). Genotypes were then phased and imputed on the TOPMed Imputation Server (https://imputation.biodatacatalyst.nhlbi.nih.gov). Variants with an imputation $R^2 > 0.8$ were retained. Samples with a mismatch between one's self-reported and genetically inferred sex, suspected sex chromosome aneuploidies, high relatedness as defined by the KING kinship coefficient (*13*)(KING > 0.177), and outlier heterozygosity (+/- 3SD from mean) were removed. Additionally, samples with a sample-level missingness > 0.05 were removed, calculated within a subset of high-quality variants (variant-level missingness ≤ 0.02).

CMC samples of EUR ancestry, as defined by assignment to the EUR superpopulation described by the 1000 Genomes Project(*30*, *33*), were isolated using a 3SD ellipsoid method. Genotypes were first merged with GRCh38 v2a 1000 Genomes Project data (https://wellcomeopenresearch.org/articles/4-50) (*33*) using BCFtools version 1.9 (*34*). PLINK 2.0 (*35*) was then used to calculate the merged genotypes' principal components (PCs), following filtering (minor allele frequency (MAF) ≥ 0.01, Hardy-Weinberg equilibrium (HWE) p-value $\geq 1 \times 10^{-10}$, variant-level missingness ≤ 0.01, regions with high linkage disequilibrium (LD) removed) and LD pruning (window size = 1000 kb, step size = 10, r2 = 0.2) steps. An ellipsoid with a radius of 3SD, corresponding to the 1000 Genomes Project EUR superpopulation, was constructed using the first three genotype PCs. Only samples that fell within this ellipsoid (n = 923) were retained for subsequent variant-level filtering. Autosomal variants with an HWE p-value $\geq 1 \times 10^{-6}$, MAF ≥ 0.01, and missingness ≤ 0.02 were thus retained, leaving 8,914,391 variants remaining.

SCZ PRS were calculated on the MVP and CMC cohort samples using summary statistics from the Psychiatric Genomics Consortium (PGC3) SCZ GWAS as training (*36*). PRS constructed with SNPs underneath schizophrenia OCRs within specific TRDs used as training data PGC3 SCZ GWAS summary statistics that were subsetted to include only SNPs falling within TRD1, TRD6, or TRD7 (for PFC-specific scores) and TRD1 or TRD2 (for ACC-specific scores), respectively. The PRS-CS-auto method (*37*) was used to apply continuous shrinkage priors to the effect sizes from these summary statistics.

A EUR LD reference panel provided by the developers of PRS-CS was utilized (https://github.com/getian107/PRScs), which draws from UK Biobank data (*38*). The following PRS-CS default settings were used: parameter a in the γ-γ prior = 1, parameter b in the γ-γ prior = 0.5, MCMC iterations = 1000, number of burn-in iterations = 500, and thinning of the Markov chain factor = 5. The global shrinkage parameter phi was set using a fully Bayesian determination method. Individual-level SCZ PRS and TRD-specific PRS were calculated using PLINK 2.0 (*35*). SCZ PRS and TRD-specific

PRS were calculated in the MVP cohort similarly to how it was calculated above, except utilising a EUR LD reference panel drawing from 1000 Genomes Project data (*33*).

The associations between scaled (mean = 0, SD = 1) PRS and diagnostic status (SCZ vs. controls, BD vs. controls, MD vs. controls) were assessed using logistic regression. For current study samples, covariates included in the analyses were age at the time of death, age squared, the first 20 genotype PCs (computed within EUR ancestry samples only), sex, and institution (Mount Sinai, University of Pennsylvania, University of Pittsburgh, or NIMH Human Brain Collection Core). For the MVP cohort, we utilised the latest MVP Phenotypes (v21.1) and used covariates for age at recruitment, age squared, the first 20 genotypes PCs (computed within the whole cohort) and sex.

PRS regression coefficients were reported as the natural logarithm of odds (logOR) along with 95% confidence intervals, and all reported p-values are unadjusted. A variance partitioning tool (https://github.com/GabrielHoffman/misc_vp/blob/master/calcVarPart.R) was used to determine the variance in SCZ(BD, MD) diagnosis status explained by the PRS variable for each regression model. These values were then divided by the number of variants used to compute each PRS, respectively, to obtain the reported "Average variance explained per variant used in estimating PRS" metrics.

Cis regulatory landscape analysis

CRD calling

Cell-region specific covariates corrected expression matrices from four datasets were utilized for CRD analysis. The datasets included: 1) neuron PFC (391,420 OCRs X 291 samples), 2) neuron ACC (391,420 OCRs X 272 samples), 3) non-neuron PFC (260,431 X 299 samples), and 4) non-neuron ACC (260,431 X 278 samples). Since disease-specific changes were not observed in BD vs. control samples at the OCR level, the CRD analysis focused only on SCZ and control samples. The CRD calling pipeline followed the methodology described in Girdhar et.al (*39*). Briefly, the first step involved estimating hidden confounds using probabilistic estimation of expression residuals (PEER)(*40*) residualization on each of the four above-mentioned matrices separately. This was done to eliminate global noise and retain the local correlation structure of OCRs in physical proximity to each other. The retained OCR correlation structure facilitated their isolation using decorate software(*41*) in the next step.

Genome-wide CRDs were called using decorate software (version 1.0.14) with mean cluster sizes of domains (10, 25, 50, 80, 100) on 11 PEER-corrected matrices, PEERs{1, 5, 10, 15, 20, 25, 30, 35, 40, 45, 50}, for each of the four datasets separately. To filter out CRDs with weaker mean correlation values, a two-step process was implemented: 1) The OCRs per chromosome were permuted to create 10 permuted matrices. CRDs were called on all 10 permuted matrices. 2) The distribution of the mean absolute correlation (MAC) metric was created for both permuted matrices and original matrices separately, for the five cluster sizes (10, 25, 50, 80, 100). MAC is defined as the mean absolute correlation value of a CRD. We estimated the MAC cutoff by taking the MAC value at 95th percentile from the distribution separately for each cluster size. **Figure S13** shows the plot of MAC vs PEERs for each cluster size. We choose PEER25 as optimal PEER for final CRDs all across the four datasets. All CRDs at the five mean cluster sizes were retained with a MAC value greater than MAC cutoff and any overlapping CRDs were merged into one after this step.

In total, 6,706(6,625) neuronal CRDs and 4,612(4,710) non-neuronal CRDs were obtained from PFC(ACC) regions (**Fig. 3B)** which encompassed, on average, of 21.7(22.1) and 18.9(18.7) OCRs per CRD that were present in neuronal and non-neuronal CRDs in PFC(ACC) respectively, with a median

number of 6 OCRs in all four datasets (**Fig S15A** and **Table S4**). We estimated cell and region specificity of CRDs by measuring jaccard index across all pairs as shown in **Fig S15B**. To measure the odds of observing CRDs to be inside a subTADs, we ran Fisher test (*42*) by using 2 x 2 contingency table of OCRs within CRDs that are inside/outside subTADs in comparison to all OCRs that are inside/outside subTADs as shown in **Fig S15C**. To test whether the OCRs within the CRD contain three-dimensional genome interactions, we utilised CTCF ChIP-seq genomic regions from ENCODE human neural cells (*43*) (syn52264219) and quantified the density of CTCF sites in 200 bins (each bin size equals to 1 kb) around CRD boundaries (**Fig. S15D-G)** and subTAD boundaries from HiC datasets for the reference (**Fig. S15H**).

Alternatively, we measured the odds of enhancer-promoter OCRs from the ABC model within a CRD vs all OCRs in a CRD. To do this, we ran Fisher test (*42*) by using 2 x 2 contingency table of cell-region specific OCRs from ABC model (*14*) that are inside/outside cell-region specific CRDs in comparison to all cell specific OCRs that are inside/outside CRDs as shown in **Fig S15I**.

Identification of schizophrenia CRDs

In our analysis, we employed a two-stage testing approach using the stageR package (*44*) to identify significant CRDs. The process involved two distinct stages: the screening stage (stage 1) and the confirmation stage (stage 2). In the screening stage, we summarized each CRD by computing its p-value using the Sidak method(*45*), which was applied to all p-values of OCRs within that particular CRD. In confirmation stage (stage 2), we focused only on CRDs with Sidak p-values less than 0.05 from stage 1. These selected CRDs underwent individual hypothesis testing to assess the dysregulation of OCRs within them. A CRD was considered a "schizophrenia CRD" if it obtained a p-value < 0.05 in stage 2, indicating significant differences in OCR expression between the SCZ and control samples. In order to summarize the directionality of CRDs, we calculated the mean of $\log_2$FC (SCZ vs Controls) of OCRs within each CRD, comparing SCZ samples to control samples. An upregulated CRD had a mean of $\log_2$FC (SCZ vs Controls) > 0, while a downregulated CRD had a mean of $\log_2$FC (SCZ vs Controls) <0 as depicted in **Fig S14C**. Differential CRDs summary table of p-values obtained from both stage 1 and stage 2 for all the identified CRDs across the four datasets is available at syn52264219.

Assessing the link of CRDs with schizophrenia OCRs and diseased genes

In order to assess whether the diseased OCRs are more likely to be within the CRD compared to outside the CRD, we employed a generalized linear model using logistic regression. The model used the t-statistics of OCRs from differential OCRs table at syn52264219 to predict the status of OCRs inside or outside the CRD as shown in eq(1). **Figure S16B** shows the odds of OCRs inside CRD from eq(1).

$$OCRs\ status\ \sim OCRs\ t - statistics(SCZ\ vs\ controls) \qquad eq(1)$$
$$where\ OCRs\ status\ \ is\ inside\ (1)\ or\ outside\ (0)\ CRD$$

Subsequently, to examine whether the schizophrenia CRDs are indeed associated with schizophrenia OCRs, we utilized eq(2). This model aimed to predict whether the CRDs is schizophrenia CRD or not using the proportion of schizophrenia OCRs inside CRDs. We have added an offset term to control for the number of OCRs within the CRD. The prediction uses Poisson model distribution in *glm* R function. **Figure S16C** shows the odds of observing the clustered schizophrenia OCRs in SCZ CRD.

$$CRD\ OCRs\ status \sim offset(log(no\ of\ OCRs\ within\ CRD)) + prop\ of\ schizophrenia\ OCRs\ inside\ a\ CRD \quad eq(2)$$

$where\ CRD\ OCRs\ inside\ or\ outside\ SCZ\ CRD\ and\ prop\ of\ schizophrenia\ OCRs\ is\ proportion\ of\ schizophrenia\ OCRs$

Lastly, to establish a connection between the disease-associated epigenome and the disease-associated transcriptome, we tested whether schizophrenia OCRs inside the schizophrenia CRDs could be predicted using the t-statistics of SCZ associated genes using eq(3). We use differential genes analysis from the CMC consortium (*19*). The OCRs were mapped to genes using ABC mapped genes table at syn52264219. **Figure S16E** shows the odds of observing the schizophrenia OCRs inside CRDs using the t-stats of differential genes.

$$schizophrenia\ OCRs\ status \sim ABC_{OCR\ genes}\ t - statistics \quad eq(3)$$

$$where\ schizophrenia\ OCRs\ status\ is\ inside\ (1)\ or\ outside\ (0)\ schizophrenia\ CRD$$

Clustering of schizophrenia CRDs into TRDs

In this section, we evaluate whether the interaction between schizophrenia CRDs across samples can stratify the CRDs into domains called trans-regulatory domains (TRDs) that can inform us about the specific molecular mechanism. We have limited the identification of TRDs to only schizophrenia CRDs in neuron PFC and neuron ACC because no significant association of CRDs with SCZ risk loci was observed in non-neurons ACC and PFC CRDs. The workflow from schizophrenia CRDs to TRDs is explained in schematic in **Fig. S17A**.

First, to obtain the interaction between schizophrenia CRDs, we took covariate corrected matrices of OCRs within neuron PFC and neuron ACC with dimensions of 1,953 x 360 and 1,691 x 330 respectively. Every CRD is summarized by calculating the average expression of OCRs using the eq(4) below:

$$CRD\ expression = 1/n \sum_{i=1}^{n} OCR_i\ where\ n = number\ of\ OCRs\ in\ a\ CRD \quad eq(4)$$

We took the Pearson correlation of CRD expression matrices that gives 1,953 x 1,953 and 1,691 x 1,691 dimensions of neuron PFC and ACC respectively. We used the first three principle components of correlation matrices for running clustering algorithm. We then fitted these matrices to hierarchical clustering with k=2:20 using diceR package in R using the default method of distance i.e. average linkage (*46*).

For every value of *k*, a random subsampling of 80% of the CRD correlation matrix is carried out 20 times. Therefore, not every sample is included in each clustering. The clustering for each iteration of the hierarchical clustering is completed using k-nearest neighbor (*47*) and majority voting. The optimal number of clusters was found by evaluating Baker-Hubert GAMMA index (*48*) while varying the cluster size (from k=2:20). GAMMA index is a measure of compactness (how similar are the objects within the same cluster), separation (how distinct are objects from different clusters), and robustness

(how reproducible are the clusters in other datasets). Using GAMMA index, we found k=10 optimal clusters across both cortical regions for neuron CRDs as shown in **Fig. S17B**. The list of annotated TRDs to CRDs from the hierarchical clustering at k=10 clusters is available at syn52264219. For every CRD, we estimated its directionality using eq (5). We added these annotations of TRDs and their direction from eq (5) to the heatmap of correlation matrix of schizophrenia CRDs in **Fig 3D** and **Fig. S18A.** Also, we curated two lists, one with all OCRs and second one with only schizophrenia OCRs within each TRDs for all downstream analysis. **Fig. S17C** shows the number of all OCRs and schizophrenia OCRs within each TRDs whereas **Fig. S17D** is stratified by directionally of SCZ OCRs within TRDs. For **Fig. 3H** and **Fig. S18E,** we took all OCRs within TRD1 to measure the spearman correlation with fetal associated changes detected at FDR < .05 in Rahman et.al. (*49*). For the full differential OCRs table of Fetal cortical ATAC OCRs vs. Adult controls OCR analysis, see syn52264219.

$$CRD\ Fold\ Change\ = 1/n \sum_{i=1}^{n} log_2FC\quad OCR_i\ eq(5)$$

$$CRD\ Fold\ Change\ >\ 0\ is\ an\ upregulated\ CRD$$

$$CRD\ Fold\ Change\ <\ 0\ is\ a\ downregulated\ CRD$$

Fetal cortical ATACseq

Sample collection

Fetal brain samples were obtained from cortical plates from four distinct donors aged 18-24 pcw. These samples were collected from de-identified prenatal autopsy specimens with no neuropathological abnormalities at the Icahn School of Medicine at Mount Sinai. Library prep and post processing of atac libraries was done similar to described above.

Association test of fetal cell types to TRDs

We obtained scATAC-seq expression data from human fetal cortical samples, as outlined in the study by Trevino et.al (*50*). The dataset comprises four samples collected over an 8-week period during mid-gestation, specifically at 16pcw, 20pcw, 21pcw, and 24pcw, containing 6423, 4486, 12675 and 7720 cells. This dataset contains 31,304 nuclei annotated to a total of 22 cell-type clusters, including five types of interneurons (IN), nine different clusters of cortical excitatory neurons (GluN) and precursor cells, such as early radial glia, late radial glia and other non-neuronal cell types. In total, there were 657,930 fetal OCRs genomic regions identified in this dataset. Only the cell types with at least 100 cells in the cell type cluster were considered for this analysis.

To assess the expression score of schizophrenia OCRs within TRDs in fetal cortical cell types, we compiled a list of schizophrenia OCRs from both TRDs and outside TRDs that overlapped at least 99% with the fetal OCRs in both cortical regions. As a result, we obtained the majority of regions for TRD1 and outside TRDs, with neuron TRD1 comprising 3,189(2,490) regions from the PFC(ACC), while outside TRDs encompassed 2,055(1,383) regions from PFC(ACC). In contrast, the number of regions that overlapped with schizophrenia OCRs in TRDs was limited, with only 263(88) regions identified in neuron PFC(ACC) TRDs(2:10). To analyze the data further, we utilized the clustering annotations provided by Trevino et.al and computed the expression scores per cluster/cell types using Seurat's

AddModuleScore function. For better visualization, we did Z-score normalization on these module scores and generated plots, as shown in **Fig 4A** and **Fig S19A**.

To investigate significant cell types associated with expression scores from our input list, our approach was two fold. First we identified TRDs that exhibited a positive and significant association with a particular cell type, relative to other cell types. To achieve this, we applied a linear mixed model, considering the enrichment scores for each TRD at different developmental stages (pcw16, pcw20, pcw21, pcw24). The model was formulated as follows: (enrichment scores ~ cell type + (1|sample ID)), where enrichment scores were obtained from the *AddModuleScore* R function(*51*). The cell type was coded as "cell type " for cells in a given cluster and "non_celltype" for cells in other clusters, and sample ID represented the individual ID of cortical samples. By incorporating the sample ID as a random effect in the model, we accounted for the presence of multiple cells from each individual. We ran this model on all cell type scores to examine their associations. Finally, we created the list of cell types and associated TRDs that had significant and positive association after FDR correction for multiple testing burden from the number of tests.

In the second step, we ran another model to compare the enrichment scores of a given cell type from a significantly associated TRD with the enrichment scores of the same cell type from schizophrenia OCRs outside the TRD. The model was defined as lmer(enrichment scores ~ TRD_OCRs_status + (1|Sample.ID) + (1|Cell.ID)), where enrichment scores were obtained as previously explained, and TRD_OCRs_status was coded as "TRD" for enrichment scores of cells from a significant TRD in a given cell type and "outside_TRD" for cells from outside TRD in the same cell type. The sample ID and cell ID represented the individual ID and cell IDs of cortical samples, respectively. We incorporated the cell ID and sample ID as a random effect to account for the repeated measurements from individuals and cells from the outside TRD enrichment scores. **Table S7A-B** and **Table S7C-D** show the output from step1 and step 2 on neuron PFC(ACC) TRDs. **Fig. 4B-C** and **Fig S19B-C** show the distribution of enrichment scores coloured by the magnitude of $\log_2$FC which is the enrichment score of schizophrenia OCRs in TRD1 in a given cell type vs enrichment score schizophrenia OCRs in TRD1 in other cell types from step 1. The star (*) annotation next to distribution shows whether or not a given cell type is significant after step 2. Our analysis showed significant association of schizophrenia OCRs in TRD1 with early glutamatergic cell types: Glu(1,3,5,8) and late glutamatergic cell types Glu(6,9) across both cortical regions. In inhibitory neuron class, IN(3,4,5) and IN(2,3,4,5) significant association with schizophrenia OCRs in TRD1 from PFC and ACC respectively. Next we gathered the cell specific markers from (*50*) for all significant cell types and intersect with ABC schizophrenia OCR mapped genes in TRD1 to obtain the cell-region and disease specific genes (see **Table S7G-H**). We used this list for running magma and pathway analysis as explained below to generate **Fig 4D-E** and **Fig. S19D-E.**

LD score enrichment analysis of CRDs

We ran LD score enrichment analysis to estimate the enrichment of brain-related and non-brain-related GWAS in all input OCR regions. For **Fig 2**, we did this analysis on all neuronal and non-neuronal OCRs using differential OCRs table (syn52264219), schizophrenia OCRs from neuron PFC, neuron ACC, non-neuron PFC and non-neuron ACC given (syn52264219). For **Fig 3**, we estimated SCZ heritability using schizophrenia OCRs within neuron PFC and neuron ACC CRDs, and outside neuron PFC and neuron ACC CRDs given in **Table S3, S6, S8.** In **Fig 4**, we did this analysis on OCRs within TRD1:10 from neuron PFC and ACC using **Table S7.** For all analyses, the broad MHC-region (chr6:25-35MB)

was excluded due to its extensive and complex LD structure, but otherwise default parameters were used for the algorithm.

MAGMA association trait analysis

We used MAGMA version 1.08b (*52*) for all the analysis described below. For **Fig. 4E-F** and **Fig. S19E-F** we took the gene markers list obtained from the above section named as "Association test of scATAC from fetal cell types to TRDs"**. Table S7G-H** tabulates cell-region and disease specific genes markers that were utilized to identify enrichment of GWAS brain and non-brain related traits in cell-region and disease specific genes. For each gene and trait, MAGMA calculates gene-level P-value based on the joint association of all SNPs from summary statistics that fall to the gene region while it accounts for linkage disequilibrium (LD) between SNPs. The gene regions were defined with the window size of 35kb upstream and 10kb downstream and LD was estimated from the European panel of 1000 Genome Project phase 3 (*33*). Then, MAGMA uses a linear regression framework to test whether the differentially expressed genes are more significantly associated with GWAS traits compared to the rest of the genome. In concordance with ATAC-seq GWAS analysis, we excluded genes that overlap the MHC-region(chr6:25-35MB).

Pathway analysis of OCRs

To functionally interpret the OCRs within TRD1 that overlapped with fetal specific OCRs (*49*) we used the Genomic Regions Enrichment of Annotations Tool (GREAT) approach to identify the biological function of nearby genes for regions using GREAT as shown in **Fig. 4F** and **Fig. S19F**. To interpret the biological function of cell-region and disease specific genes from section "Association test of scATAC from fetal cell types to TRDs", we applied GREAT pipeline to obtain **Fig 4D**, **Fig S19D** using the **Table S7G** and **Table S7H** respectively**.**

Captions for Supplementary Figures S1-S21

Figure S1 | Summary metadata of samples in the study.
Figure S2 | Quality control metrics in cell- and region-specific samples stratified by diagnosis.
Figure S3 | Quality control metrics of neuronal and non-neuronal consensus OCRs.
Figure S4 | Variance explained by identified covariates in normalized counts of samples for all OCRs.
Figure S5 | Annotation of OCRs to regulatory elements and similarity of OCRs with other datasets.
Figure S6 | Annotation of OCRs to genes using the Activity-By-Contact (ABC) method.
Figure S7 | Covariate correction of the expression of cell specific OCRs.
Figure S8 | Mean variance distribution of normalized counts of neuronal and non-neuronal OCRs.
Figure S9 | Correlation of SCZ and BD specific OCRs.
Figure S10 | LDsc enrichment of cell and region specific schizophrenia OCRs.
Figure S11 | Inferred SCZ stages from chromatin accessibility generated from ACC in neurons.
Figure S12 | Distribution of technical and clinical variables of samples stratified by inferred disease stages.
Figure S13 | Optimal number of PEER to call final CRDs.
Figure S14 | *Cis*-regulatory landscape defined by CRDs.
Figure S15 | Higher order chromatin structure related characteristics of CRDs.
Figure S16 | Disease relevance of CRDs.
Figure S17 | Characteristics of trans-regulatory domains of CRDs.
Trans regulatory domains from hierarchical clustering of neuronal SCZ CRDs from the ACC
Figure S18 | Trans regulatory domains from hierarchical clustering of neuronal SCZ CRDs from the ACC.
Figure S19 | Analysis of cell-type specificity of neuronal ACC schizophrenia OCRs in fetal cortical scATAC-seq data.
Figure S20 | Enrichment of expression of schizophrenia OCRs in SCZ TRDs in fetal cortical OCRs in scATAC-seq data.
Figure S21 | Trans Regulatory Domain one (TRD1) in PFC and ACC can stratify SCZ cases and controls in MVP and CMC cohort.

Supplementary Figures S1-21

Region	Cell types	Diagnosis	Sex	Ancestry	Age	Brain Bank
PFC	Neuron	102 (SCZ)<br>69 (BD)<br>189 (Ctrl)	228 M<br>132 F	242(EUR)<br>111(AA)<br>4(HIS),3(A)	55.9±20.8	147(MSSM)<br>154(HBCC)<br>59(Pitt)
	Non-neuron	107 (SCZ)<br>68 (BD)<br>192 (Ctrl)	233 M<br>134 F	251(EUR)<br>111(AA)<br>3(HIS), 2(A)	56.1±20.7	149 (MSSM)<br>157(HBCC)<br>61(Pitt)
ACC	Neuron	98 (SCZ)<br>58 (BD)<br>174 (Ctrl)	206 M<br>124 F	233(EUR)<br>94(AA)<br>1(HIS), 2(A)	57.7±20.2	139 (MSSM)<br>128 (HBCC)<br>63 (Pitt)
	Non-neuron	99 (SCZ)<br>58 (BD)<br>179 (Ctrl)	212 M<br>124 F	237(EUR)<br>94(AA)<br>3(HIS), 2(A)	57.5±20.3	142(MSSM)<br>129(HBCC)<br>65(Pitt)

SCZ: Schizophrenia, BD: Bipolar Disorder, Ctrl: Control
M: Male, F: Female
EUR: European, AA: African-American, HIS: Hispanic, A: Asian
MSSM: Mount Sinai NIH Brain Bank and Tissue Repository
HBCC: NIMH Human Brain Collection Core
Pitt: University of Pittsburgh NIH NeuroBioBank Brain and Tissue Repository.

Figure S1 | Summary metadata of samples in the study.

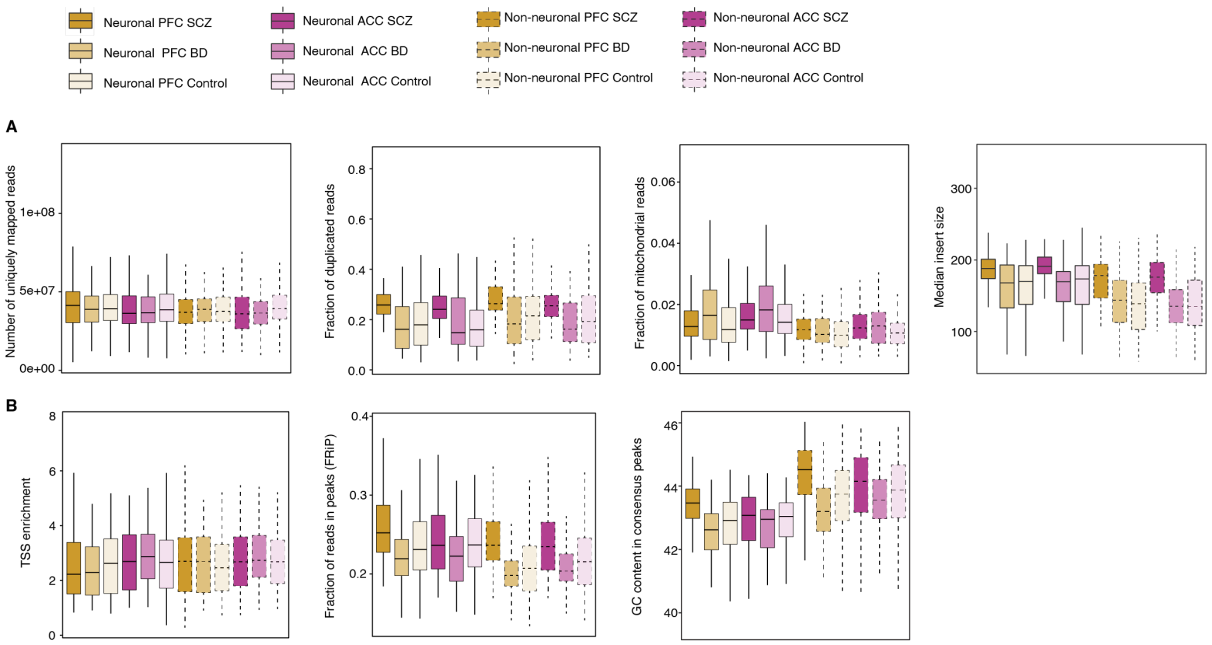

Figure S2 | Quality control metrics in cell- and region-specific samples stratified by diagnosis. A) Distribution of uniquely mapped reads, fraction of duplicated reads, fraction of mitochondrial reads, and median insert size for each sample. **B)** Distribution of TSS enrichment of OCRs using all housekeeping genes from Refseq (*9*), fraction of reads in OCRs, and gc content in OCRs for each sample. In both A) and B), box plots are depicted with the center line representing the median, the box indicating the interquartile range (between the 25th and 75th percentile), and whiskers extending to the lowest and highest values within 1.5 times the interquartile range from the median.

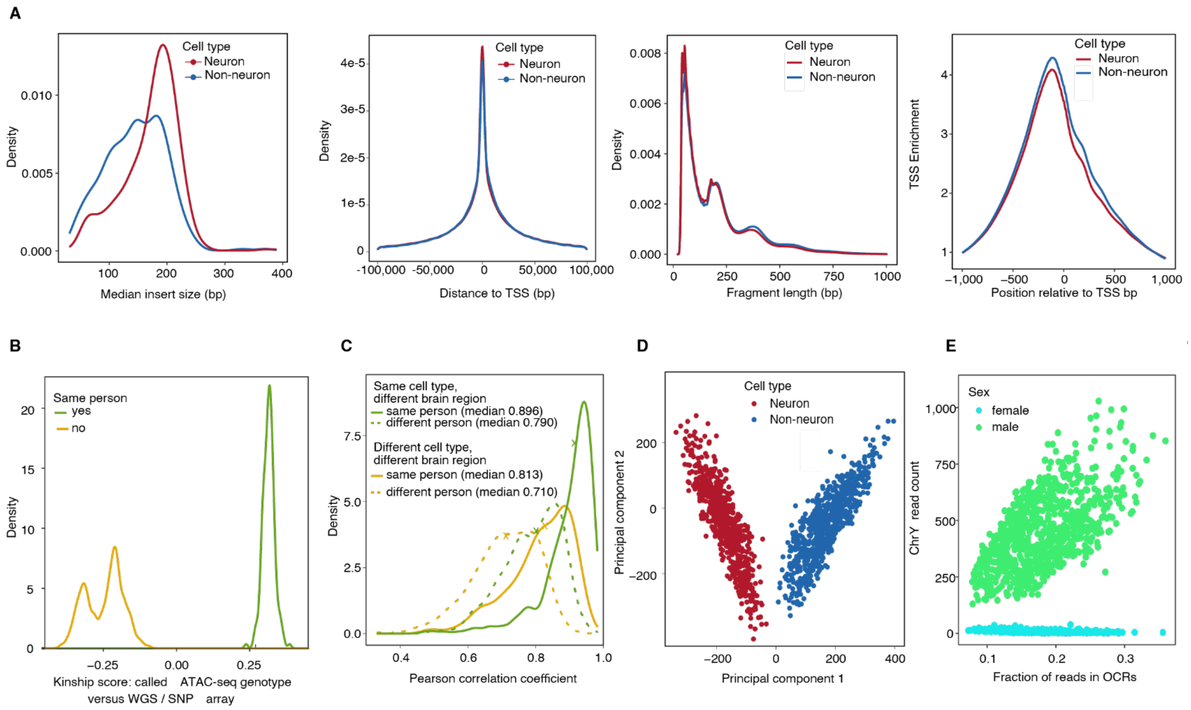

Figure S3 | Quality control metrics of neuronal and non-neuronal consensus OCRs. **A)** Distribution of median read insert size, distance to TSS, fragment length, and TSS enrichment of cell-specific consensus OCRs. **B)** Genotype checks were performed using pairwise comparisons of genotypes called from ATAC-seq with genotypes from whole-genome sequencing, as well as with the ATAC-seq libraries themselves. **C)** Correlations of raw reads coverage (number of reads) over consecutive bins of 10 kb genomic regions between samples originating from the same versus different person (yellow line: samples originating from the same cell type but different brain region; green line: samples originating from different cell types but the same brain regions). Median correlations are highlighted by the “x” symbol." **D)** Principal component analysis (PCA) was applied to evaluate chromatin accessibility levels in OCRs, with cell types represented in red for neuronal and blue for non-neuronal cells. **E**) Sex check based on measuring the number reads mapped on chromosome Y.

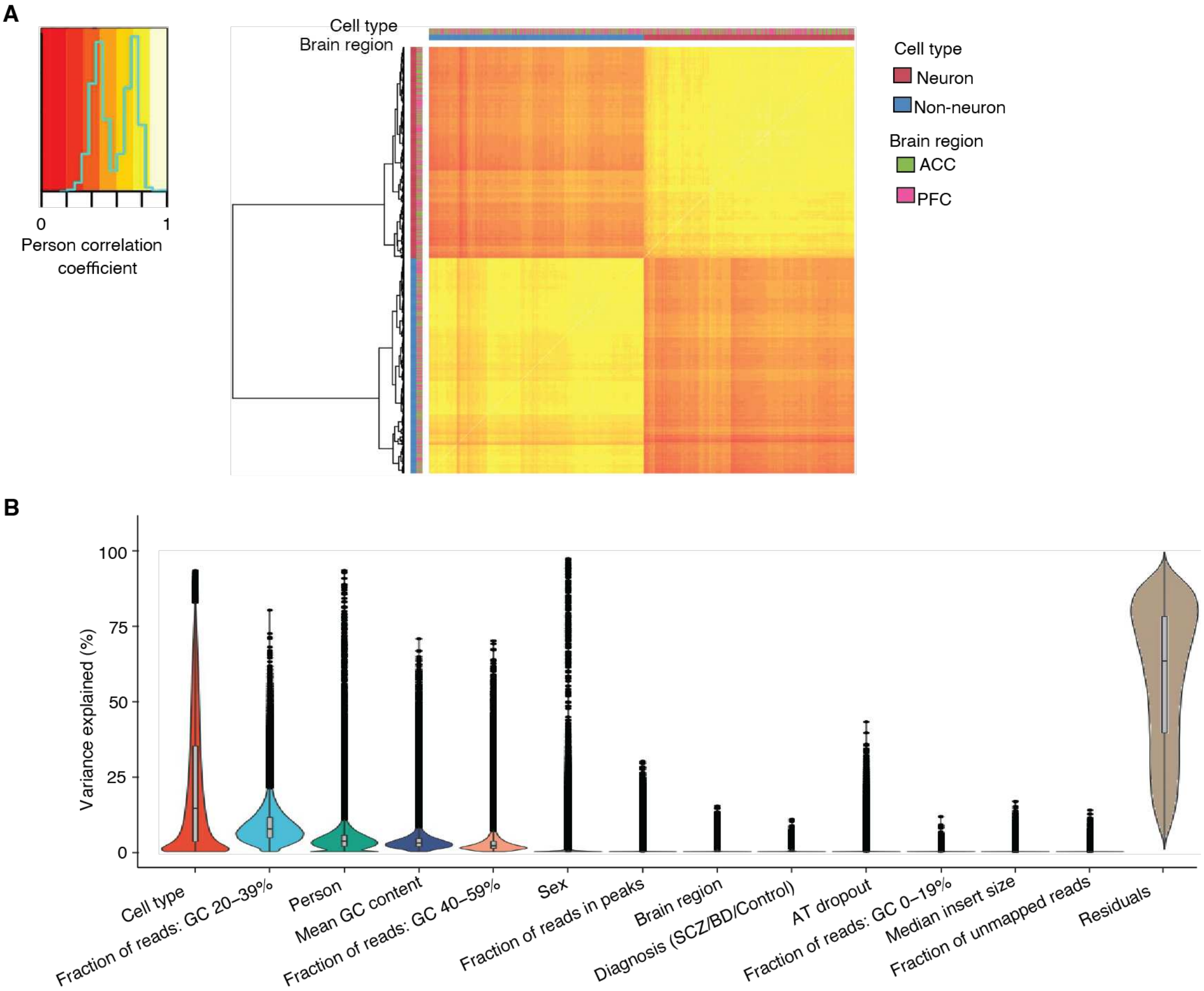

Figure S4 | Variance explained by identified covariates in normalized counts of samples for all OCRs. A) Heatmap representing the Pearson correlation of expression levels for merged chromatin regions across all samples. The bar above the heatmap displays cell and region type labels corresponding to the samples. **B)** Distribution of variance explained by each OCR with respect to different covariates. The covariates on the x-axis were selected using a BIC model explained in the methods section. Box plots are depicted with the center line indicating the median, the box representing the interquartile range (between the 25th and 75th percentiles), and whiskers extending to the lowest and highest values within 1.5 times the interquartile range from the median. Dots are used to indicate potential outliers beyond this range.

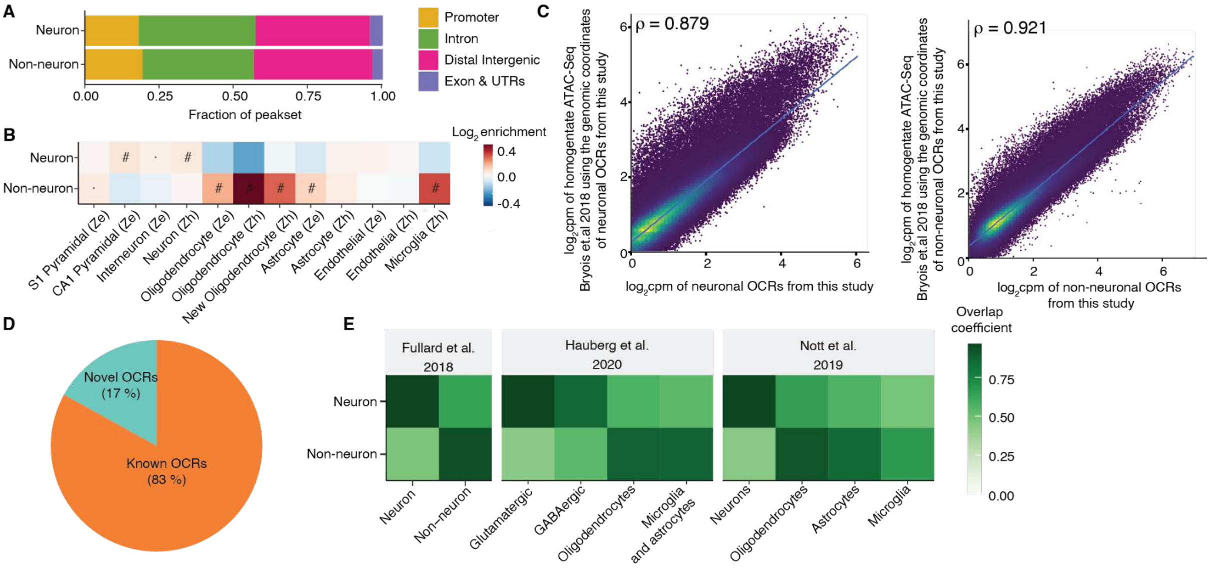

Figure S5 | Annotation of OCRs to regulatory elements and similarity of OCRs with other datasets. A) Bar plot depicting the percentage of regulatory elements in each cell-specific OCR, obtained after annotation using ChIPseeker. **B)** Enrichment analysis showing the overlap of cell-specific OCRs with cell-specific marker genes using the resource from 'Ze' (Zeisel)(*53*) and 'Zh' (Zhang)(*54*). '#': significant for enrichment after FDR correction of multiple testing across all tests in the plot (Benjamini–Hochberg test); '·': nominally significant for enrichment. **C)** Spearman correlation analysis illustrates the expression count ($\log_2$cpm) correlation of neuronal OCRs from samples in this study with expression counts ($\log_2$cpm) of samples from PFC homogenate in Bryois et al.'s (*55*) study. **D)** Pie plot displaying the distribution of OCRs categorized as known (overlapping with other studies(*2*, *3*, *55–59*)) and novel (unique to this study). **E)** Overlap coefficient to show the overlap of OCRs identified in this study with cell-specific OCRs from other published datasets(*3*, *56*, *57*).

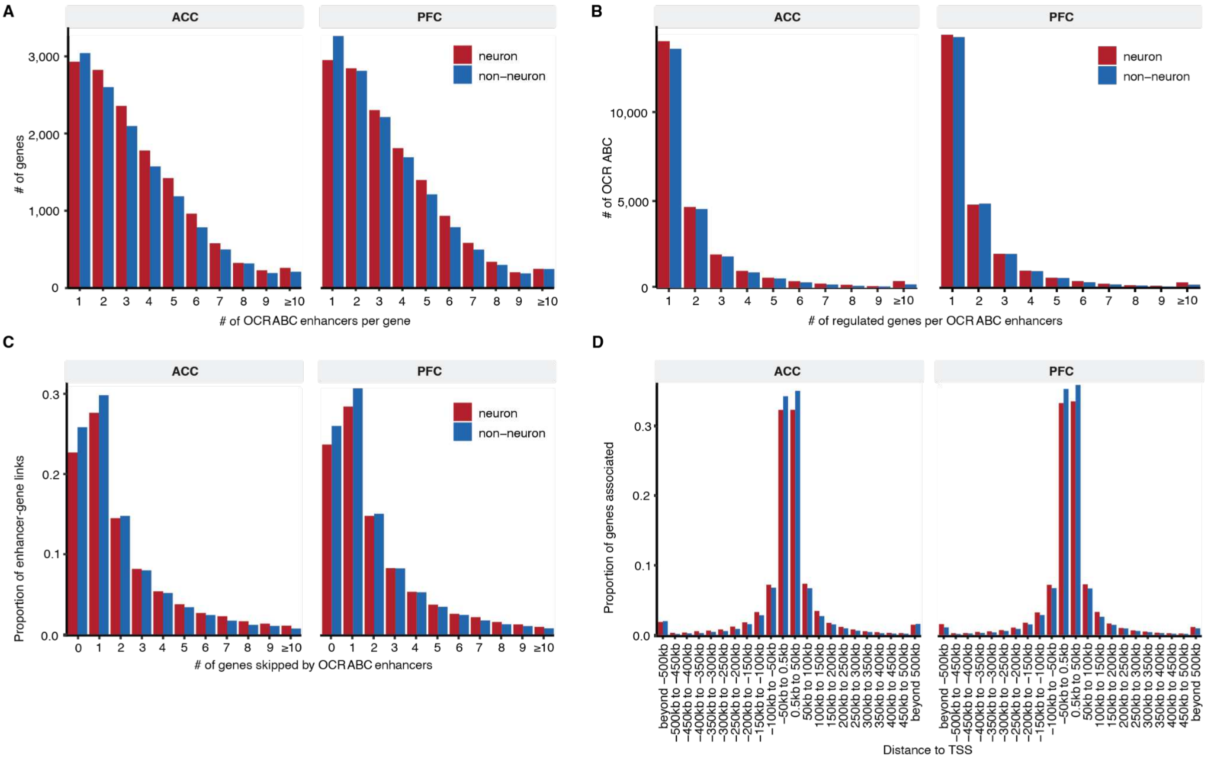

Figure S6 | Annotation of OCRs to genes using the Activity-By-Contact (ABC) method. **A**) Histogram of the number of OCR_{ABC} linked per gene. **B**) Histogram of the number of genes linked per OCR_{ABC}. **C**) Histogram of the number of genes "skipped" by an OCR_{ABC} to reach their linked genes. **D**) Histogram of the distance of OCR_{ABC} to the TSS of regulated genes.

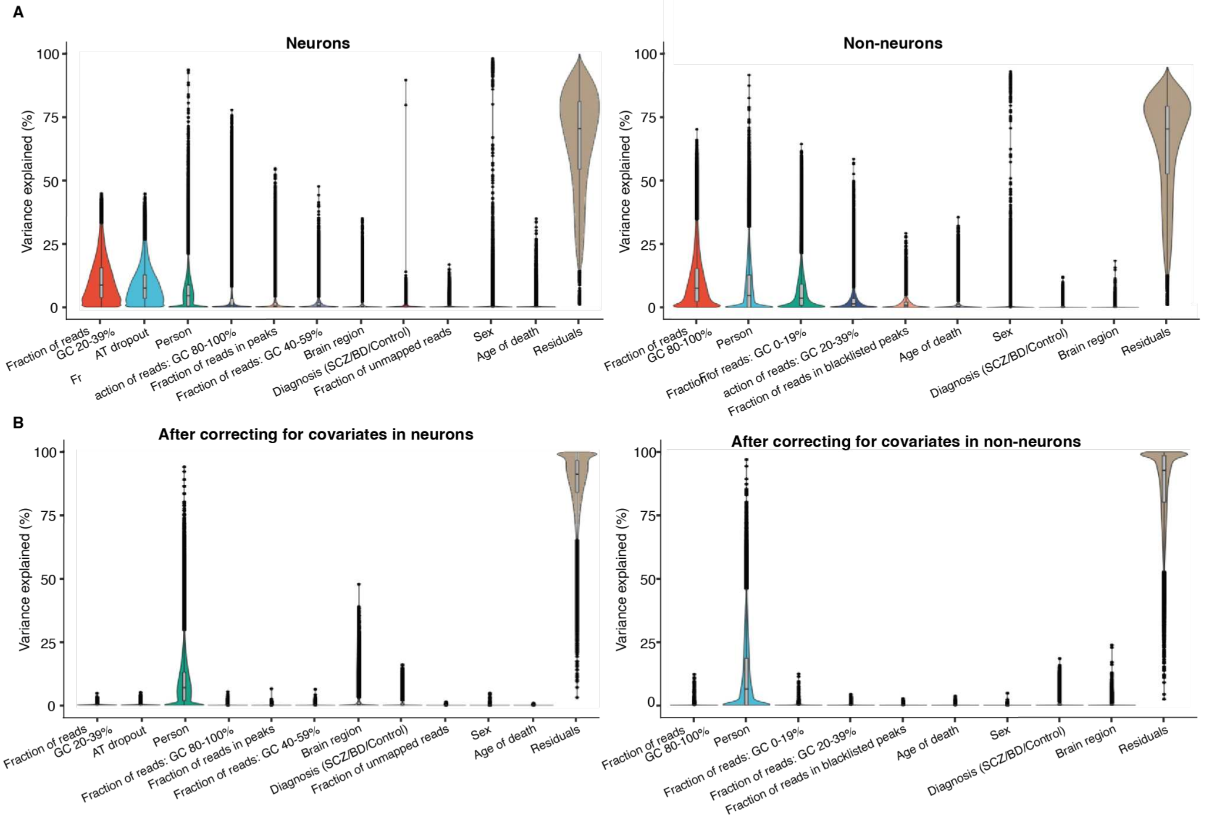

C

Neurons

Variable	Variance explained [%] Pre-covariate adjustment	Post-covariate adjustment
Fraction of reads with GC 20-39%	10.52	0.20
AT dropout	8.93	0.18
Person	5.66	8.63
Fraction of reads with GC 80-100%	3.66	0.03
Fraction of reads in peaks	1.76	0.02
Fraction of reads with GC 40-59%	1.44	0.05
Brain region	0.86	1.16
Diagnosis (SC/BD/Control)	0.44	0.68
Fraction of unmapped reads	0.39	0.01
Sex	0.29	<0.01
Age of death	0.26	0.01
Residuals	65.79	89.04

Non-neurons

Variable	Variance explained [%] Pre-covariate adjustment	Post-covariate adjustment
Fraction of reads with GC 80-100%	11.12	0.08
Person	8.25	11.17
Fraction of reads with GC 0-19%	6.47	0.12
Fraction of reads with GC 20-39%	2.68	0.09
Fraction of reads in blacklisted peaks	1.57	0.04
Age of death	1.15	0.03
Sex	0.35	<0.01
Diagnosis (SC/BD/Control)	0.33	0.46
Brain region	0.26	0.35
Residuals	67.82	87.66

Figure S7 | Covariate correction of the expression of cell specific OCRs. **A)** Violin plots to show the distribution of variation of OCRs for biological and technical covariates in neurons (left) and non-neurons (right) before and **B)** after correcting for covariates. All technical covariates (i.e. except Sex, Brain region, Person and Residuals) were identified using the BIC model explained in the methods section. **C)** Mean proportion of overall variance in OCRs attributed to the biological and technical covariates before and after correcting for technical (but not biological) covariates in neurons (left) and non-neurons.

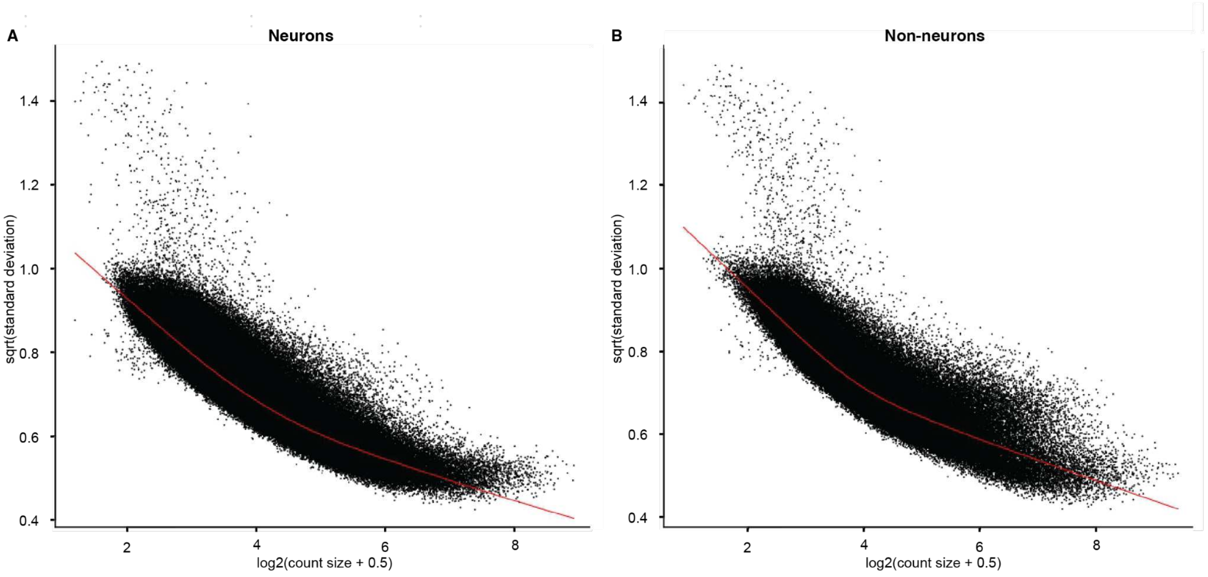

Figure S8 | Mean variance distribution of normalized counts of neuronal and non-neuronal OCRs. A) and **B)** show the plot of mean vs variance in expression of neuronal and non-neuronal OCRs shown as black dots with LOWESS trend as a red line that indicate low - moderate biological variation.

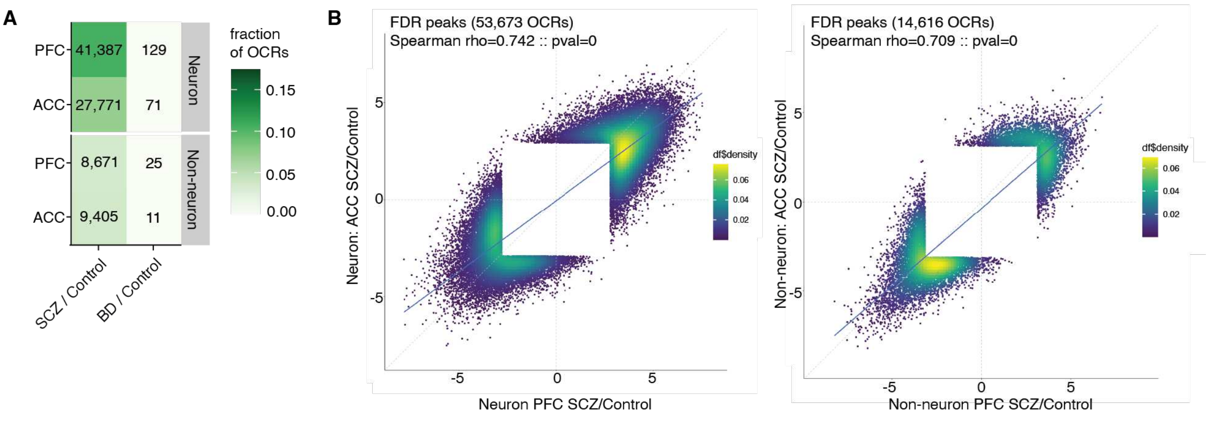

Figure S9 | Correlation of SCZ and BD specific OCRs. A) Heatmap of the number of differential OCRs at FDR < .05 in for all 4 datasets: neuron PFC, neuron ACC, non-neuron PFC and non-neuron ACC across SCZ/controls and BD/controls associated. **B)** Comparison of t-statistics of significant SCZ associated changes at FDR < .05 across cortical regions in neuronal (53,673 OCRs) and non-neuronal (14,616 OCRs). White square near the origin corresponds to the OCRs that did not pass the FDR cutoff.

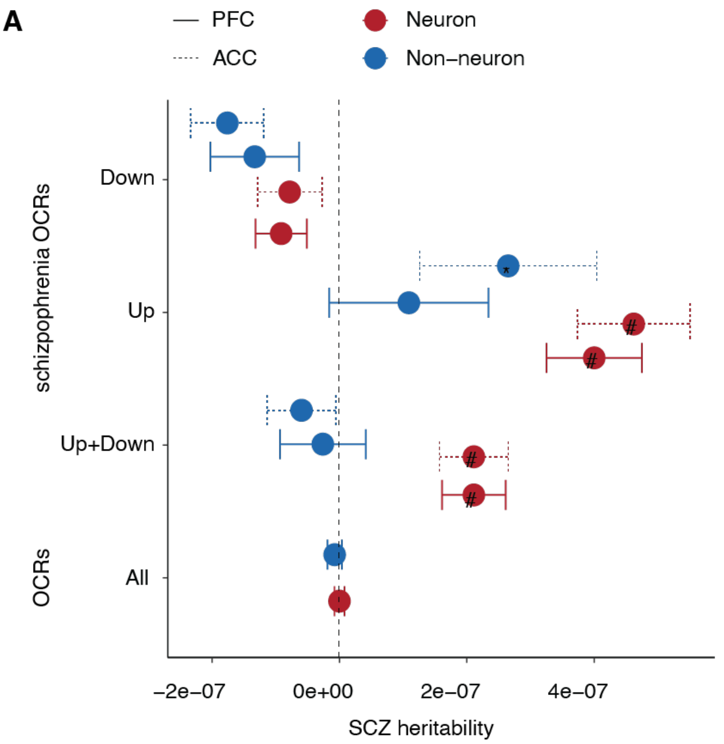

Figure S10 | SCZ heritability of cell and region specific schizophrenia OCRs. A) Schizophrenia heritability coefficients of common risk variants that overlap with (1) 'All OCRs': all identified OCRs (391,420 in neurons (red) and 260,431 in non-neurons (blue)), (2) "Up+Down": upregulated and downregulated schizophrenia OCRs (41,387(27,771) in PFC(ACC) neurons (red), 8,671(9,405) in PFC(ACC) non-neurons (blue)) (3) "Up": upregulated schizophrenia OCRs (25,146(15,791) in PFC(ACC) in neurons (red), 3,726(3,075) in PFC(ACC) non-neurons (blue)); and (3) "Down": dysregulated schizophrenia OCRs (16,241(11,980) in PFC(ACC) neurons (red), 4,945(6,330) in PFC(ACC) non-neurons (blue)). All upregulated and downregulated OCRs are with $\log_2$FC (schizophrenia versus controls) >0 and <0, respectively. Error bars represent standard error in schizophrenia heritability from LD score regression.

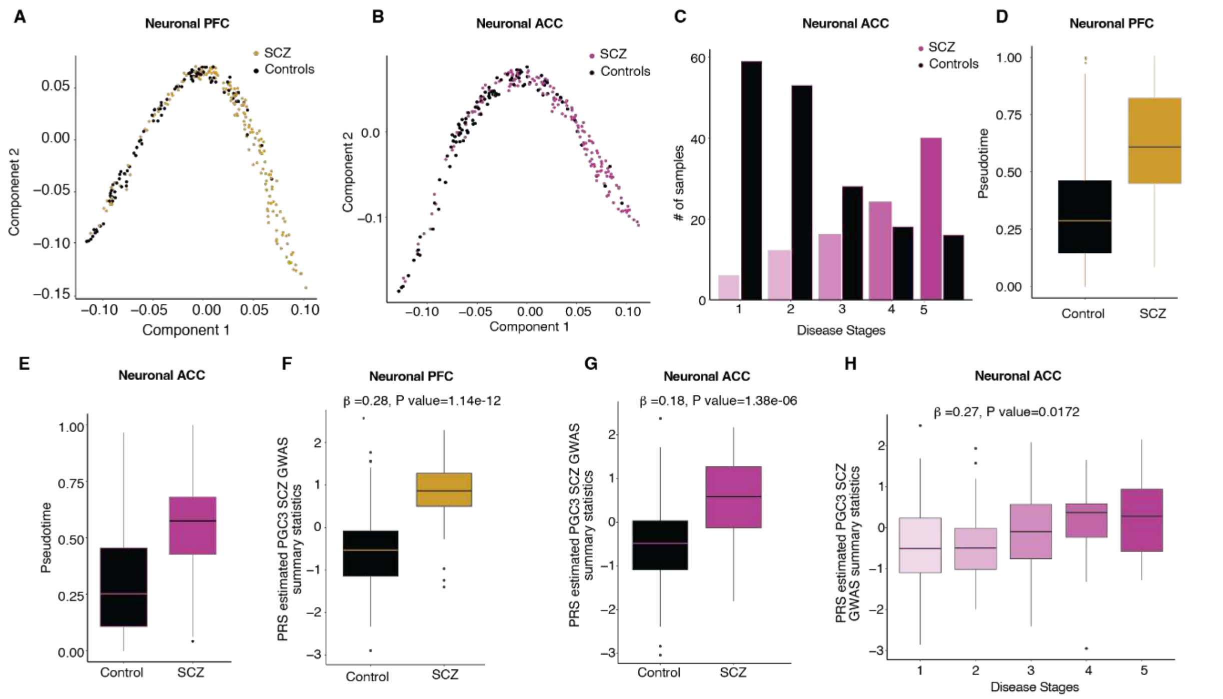

Figure S11. Inferred SCZ stages from chromatin accessibility generated from PFC and ACC in neurons. A-B) Estimated low-dimensional space to order samples based on similarity in expression of upregulated neuronal OCRs (n=2,411(1,495)) in SCZ from PFC(ACC) regions. The samples are colored by clinical diagnosis. SCZ samples are in yellow(purple) and controls are in black(black) in PFC(ACC) regions. **C)** Stratification of samples by clinical diagnosis as a function of inferred disease stage, where early stage (n=1) to late stage (n=5) is from left to right. **D-E)** Distribution of pseudotime for neuronal PFC and neuronal ACC samples in C and D respectively. Logistic regression model: schizophrenia/Controls ~ pseudotime; OR=2.9, P value = 1.69e-05 in PFC (D) and OR=2.8, P value = 9.50e-05 in ACC (E). **F-G)** Plot of PRS of samples calculated using PGC3 SCZ GWAS summary statistics, stratified by inferred disease status in PFC and ACC region respectively. Beta estimate and p value in G-H are obtained using linear regression model: disease stages/status ~ PRS + Age + Age^2 + Sex. **H)** Plot of PRS of samples calculated using PGC3 SCZ GWAS summary statistics, stratified by inferred disease severity stages in ACC region. Box plots in D-H have lower and upper hinges at the 25th and 75th percentiles and whiskers extending to, at most, 1.5xIQR (interquartile range).

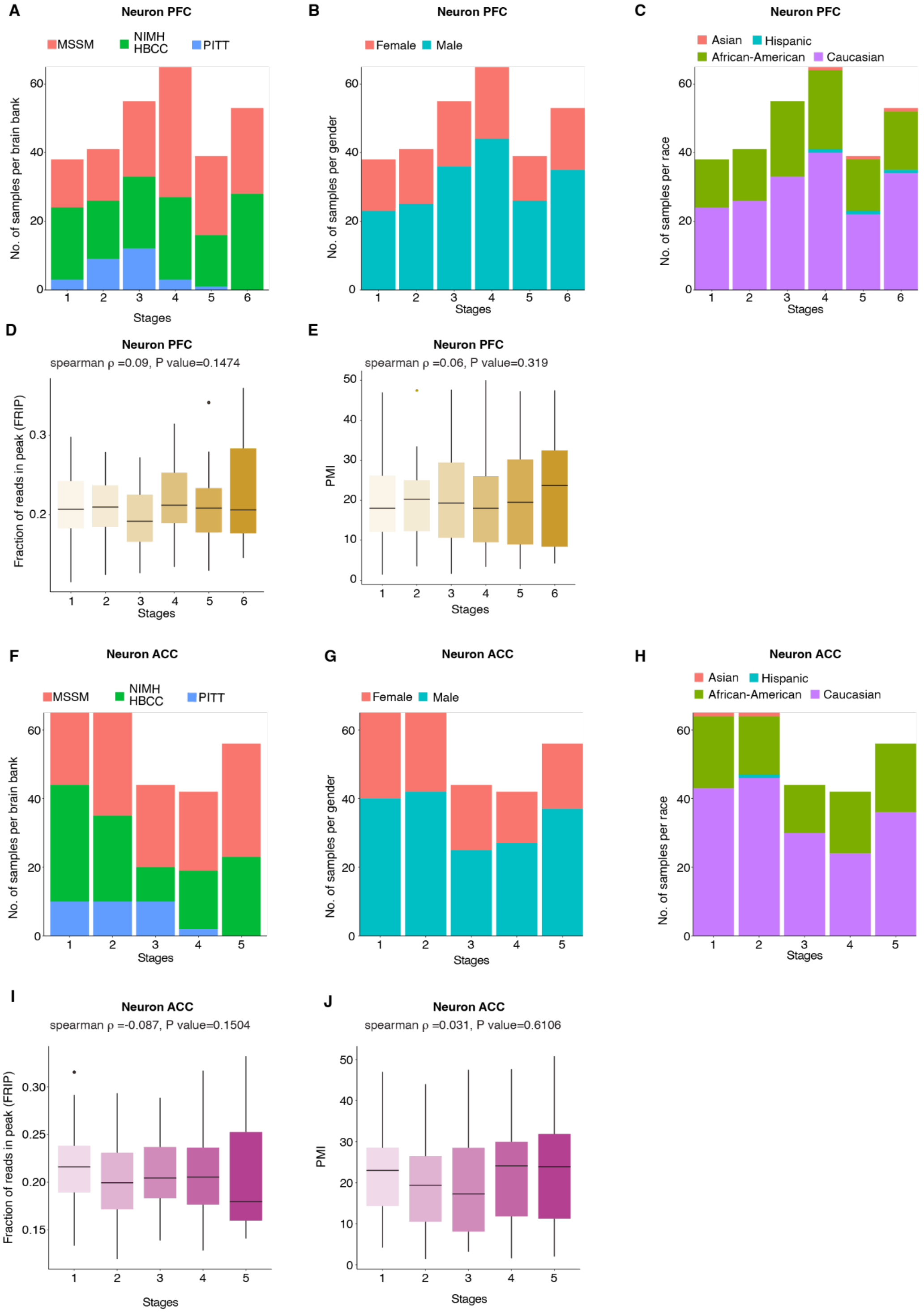

Figure S12. Distribution of technical and clinical variables of samples stratified by inferred disease stages. A-C) Bar plot to show the brain-bank, gender and race of neuron PFC samples stratified by inferred disease stages. **D-E)** Distribution of fraction of reads in peak(frip) and postmortem interval (PMI) in hrs of neuron PFC samples as a function of inferred disease stages. **F-H)** Bar plot to show the brain-bank, gender and race of neuron ACC samples stratified by inferred disease stages. **I-J)** Distribution of fraction of reads in peak (frip) and postmortem interval (PMI) in hrs of neuron ACC samples as a function of inferred disease stages. Box plots in D-E) and I-J) have lower and upper hinges at the 25th and 75th percentiles and whiskers extending to, at most, 1.5xIQR (interquartile range).

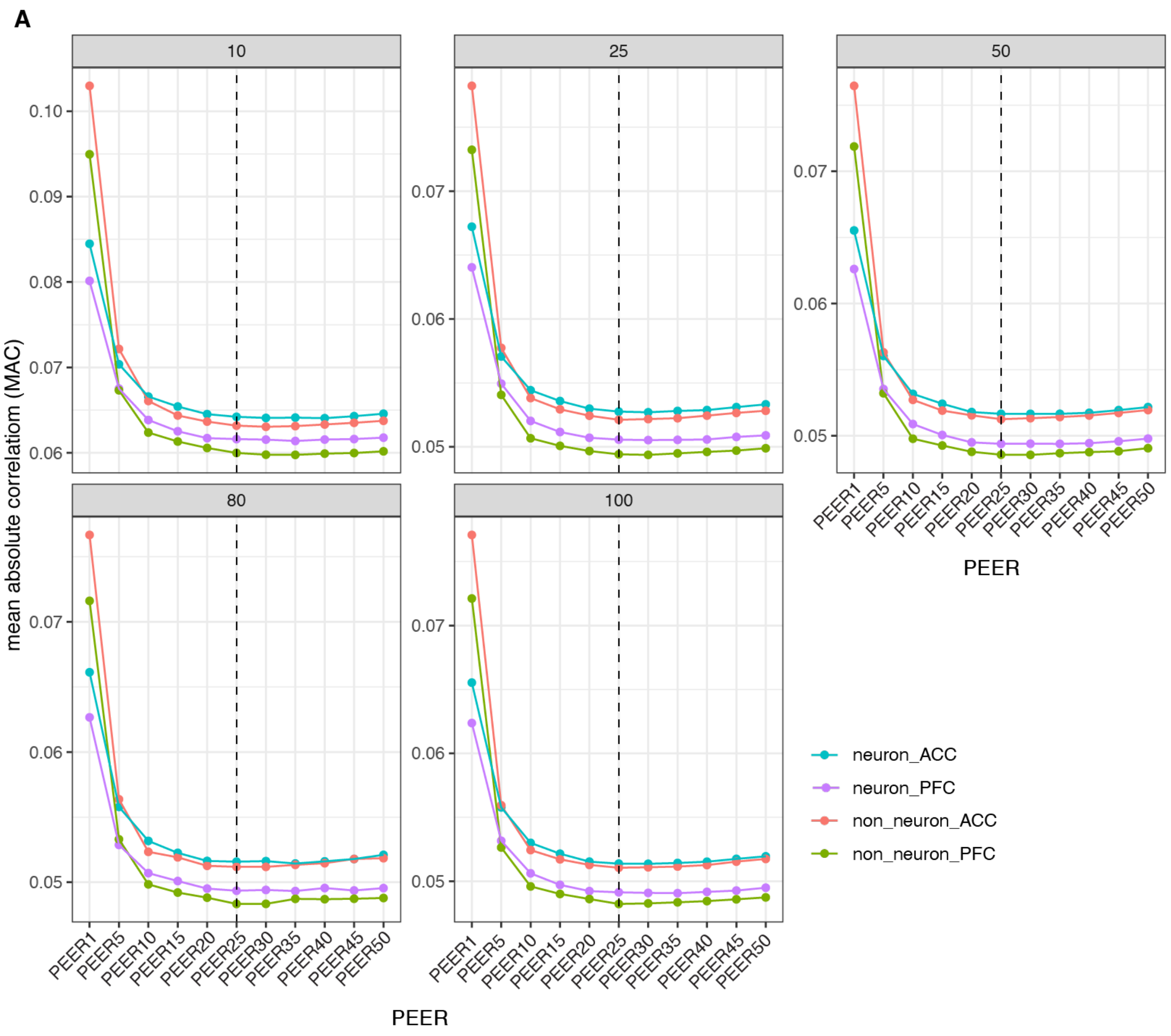

Figure S13 | Optimal number of PEER to call final CRDs. A) Plot of mean absolute correlation (MAC) cut off value which is the value at 95th percentile from MAC distribution of MAC of each CRDs and permuted CRDs at i^{th} PEER.

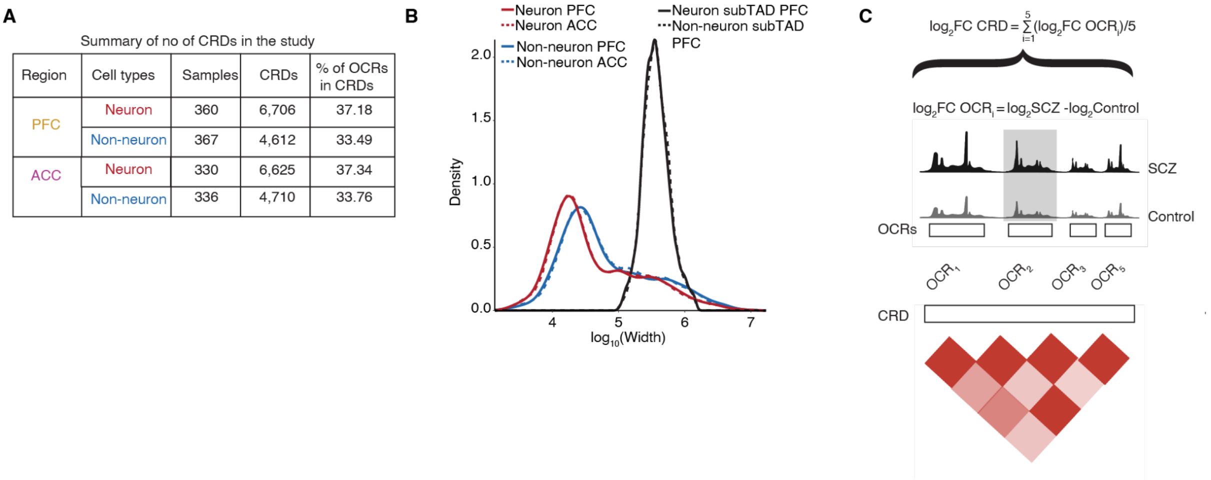

Region	Cell types	Samples	CRDs	% of OCRs in CRDs
PFC	Neuron	360	6,706	37.18
	Non-neuron	367	4,612	33.49
ACC	Neuron	330	6,625	37.34
	Non-neuron	336	4,710	33.76

Figure S14 | *Cis*-regulatory landscape defined by CRDs. A) Summary table of the number of samples, OCRs and CRDs identified across four datasets 1) neuronal PFC, 2) neuronal ACC, 3) non-neuronal PFC and 4) non-neuronal ACC. **B**) Genome-wide base pair length distribution (x-axis log scale) of CRDs (colored curves) compared to neuronal and non-neuronal PFC subTADs (black curve). **C**) Schematic of a CRD obtained from the pairwise correlation of four OCRs. The $\log_2$FC CRD is summarized as the average of $\log_2$FC (SCZ versus controls) of four OCRs.

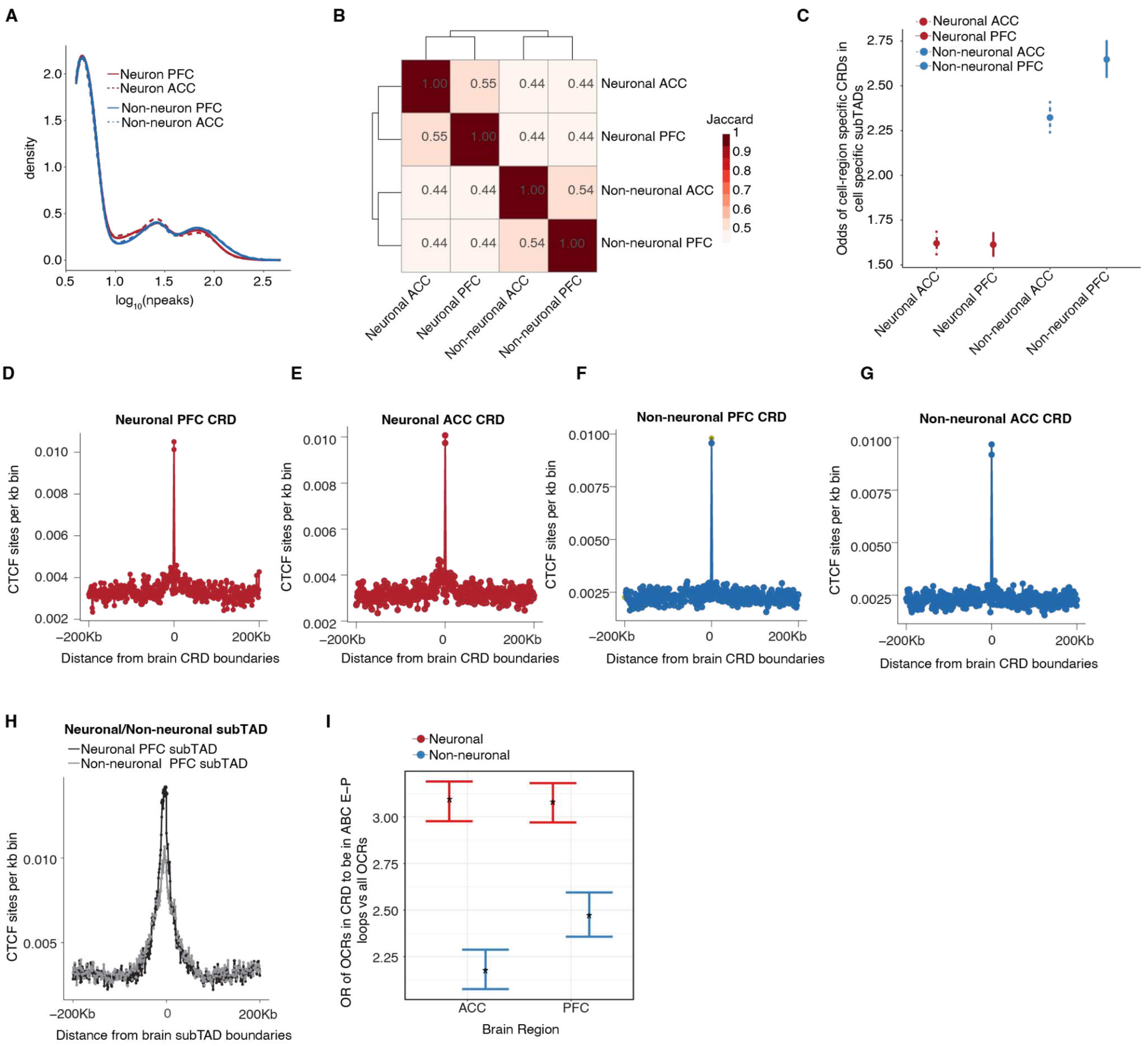

Figure S15 | Higher order chromatin structure related characteristics of CRDs. **A**) Distribution of number of OCRs per CRD in neuron PFC, neuron ACC, non-neuron PFC and non-neuron ACC CRDs. The x-axis is in log scale. **B)** Jaccard index to show the overlap of OCRs within the cell and region specific CRDs. **C)** Odds ratios to find the cell-region specific OCRs within CRDs inside PFC cell specific subTADs when compared to all OCRs. Proportion of CTCF sites in a bin of 1Kb as a function of distance from boundaries of neuronal CRD in **D)** PFC (red), **E)** ACC (red**)**, **F)** non-neuronal CRD PFC (blue), **G)** ACC (blue**)**, **H)** PFC neuron subTAD (black) and PFC non-neuronal subTAD(gray). CTCF regions are from H1 stem-cell-differentiated neuronal culture in relation to distance from CRD. **I)** Odds ratio to find ABC OCRs within neuronal PFC(red), neuronal ACC(red) and non-neuronal PFC(blue), non-neuronal ACC(blue) CRDs. (neurons: OR>3, Fisher's exact test p value < 0.05) and non-neurons CRDs (OR>2, Fisher's exact test p value < 0.05)

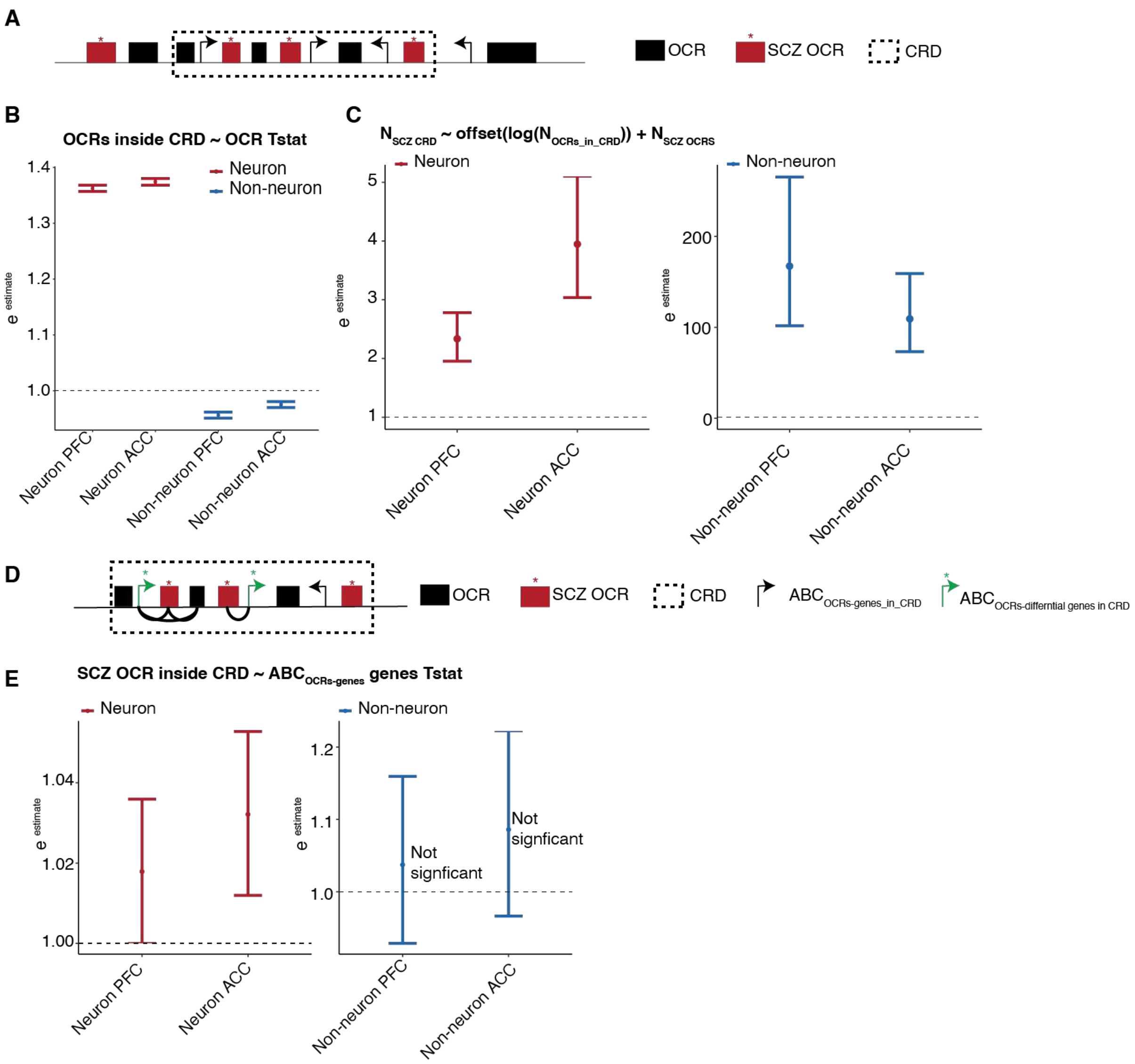

Figure S16 | Disease relevance of CRDs. A) Schematic representation showing schizophrenia OCRs in red color with a "*," while non-schizophrenia OCRs are depicted in black. An example of a CRD is enclosed within a dashed box, and black arrows indicate TSS sites of genes. **B)** Coefficient of the logistic regression-based generalized linear model used to predict the status of neuronal (non-neuronal) OCRs located inside or outside neuronal (non-neuronal) CRDs in both cortical regions colored in red(blue). This prediction is made using SCZ-associated t-statistics obtained from the cell-region specific differential OCRs analysis. **C)** Coefficient of the Poisson-based glm (generalized linear model) to predict whether a CRD is a SCZ CRD or not using the number of schizophrenia OCRs within the CRD as an independent variable. An offset term of the number of OCRs within the CRD was included in the model. **D)** Schematic representation showing schizophrenia OCRs in red with "*", and non-schizophrenia OCRs in black. An example of a CRD and OCRs within it are enclosed within a dotted box. Black arrows show TSS sites of genes mapped to OCRs, while green arrows indicate TSS sites of ABC differential genes in SCZ vs. Controls, which are mapped to OCRs. **E)** Coefficient of the logistic regression-based generalized linear model used to predict the status of neuronal (non-neuronal) schizophrenia OCRs located inside or outside neuronal (non-neuronal) CRDs in both cortical regions colored in red(blue). SCZ-associated t-statistics obtained from the differential genes mapped to OCRs within the CRD is an independent variable in the model. The differential genes analysis is of CMC cohort(*19*).

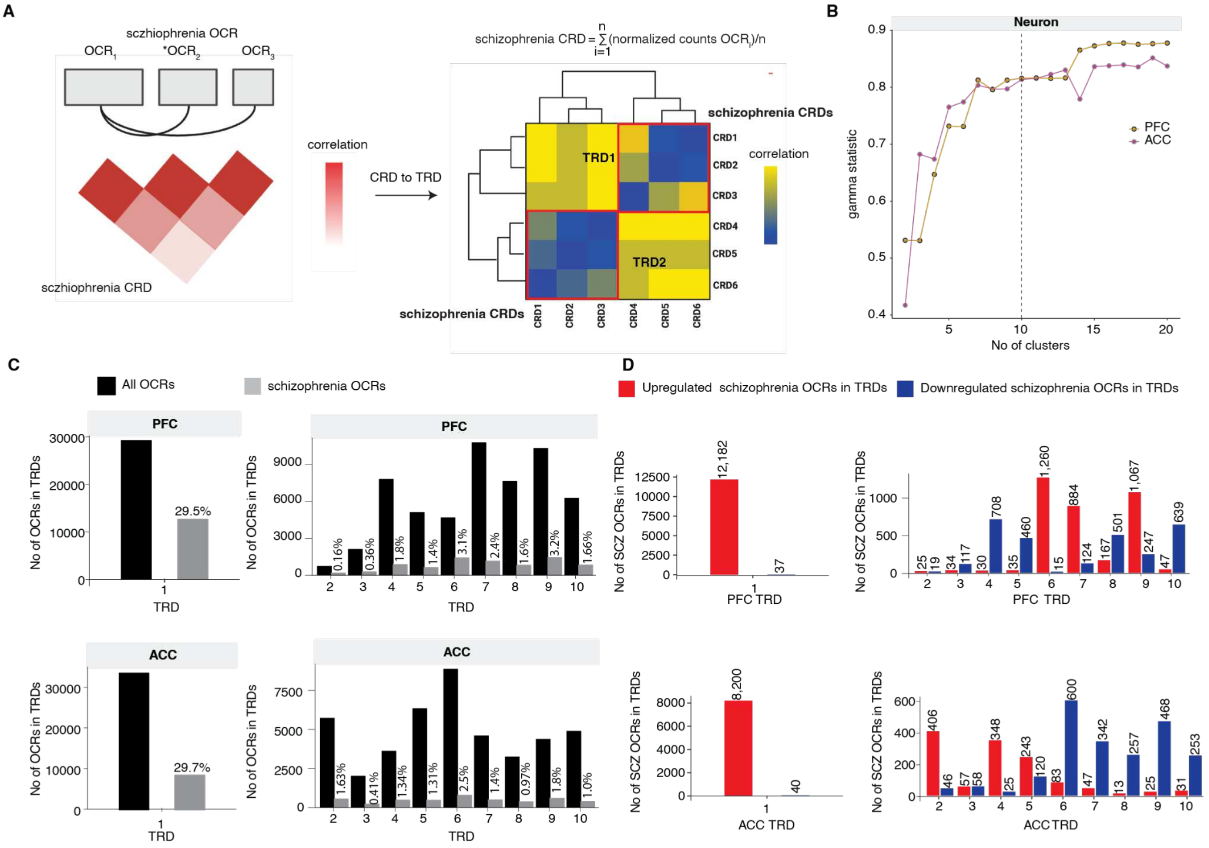

Figure S17 | Characteristics of trans-regulatory domains of CRDs. A) Schematic workflow depicting identification of TRDs from hierarchical clustering of schizophrenia CRDs. **B)** Gamma statistics were plotted against the number of clusters in Chromatin Regulatory Domain (CRD) matrices of neuronal PFC and ACC. The dotted line indicates the optimal number of clusters, *n*=10, which was selected for both neuronal PFC and ACC through the hierarchical clustering method. **C)** The number of OCRs and SCZ associated OCRs are represented as "All OCRs" and "schizophrenia OCRs," respectively, within CRDs in neuronal PFC TRDs (top plots) and non-neuronal ACC TRDs (bottom plots). **D)** The number of upregulated and downregulated schizophrenia OCRs within TRDs are depicted separately in neuronal PFC TRDs (top plots) and neuronal ACC TRDs (bottom plots).

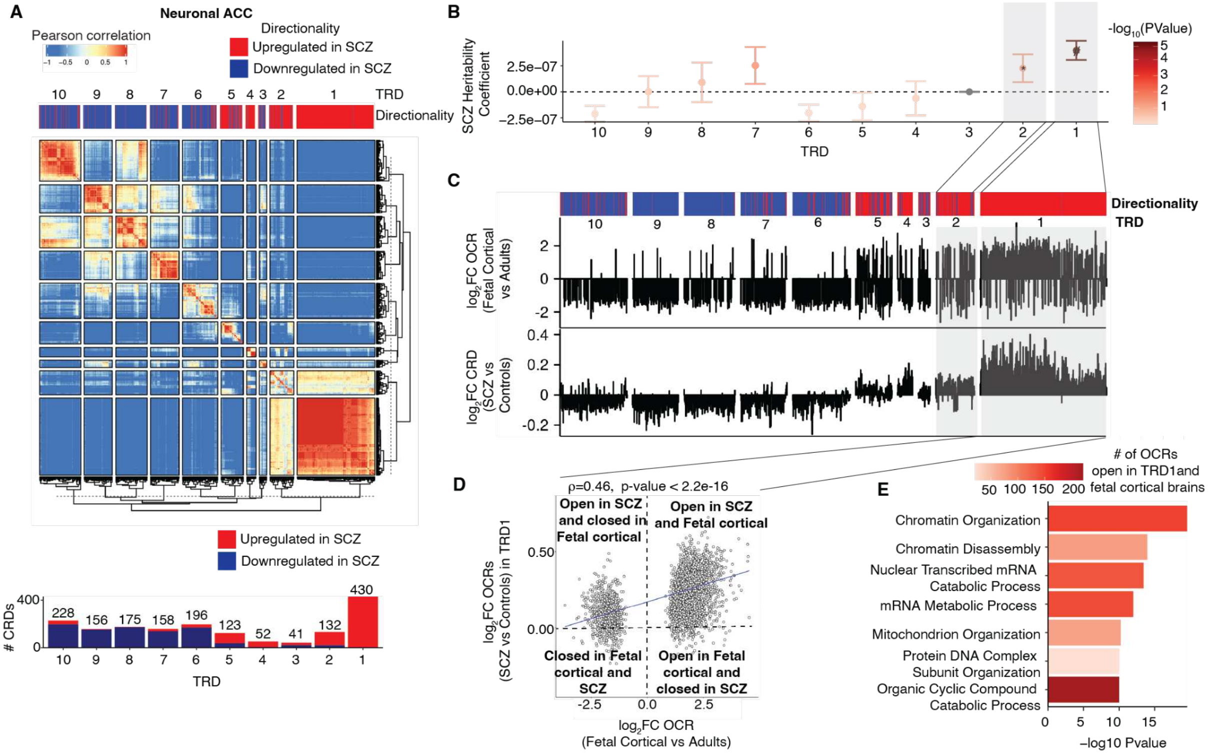

Figure S18 | Trans regulatory domains from hierarchical clustering of neuronal SCZ CRDs from the ACC. (A) Heatmap depicting hierarchical clustering of trans interaction of 1,691 SCZ CRDs across 330 samples from the ACC. The clustering results in ten trans regulatory domains (TRDs) using gamma statistics. The directionality annotation bar plot above the heatmap shows upregulation in red (log_2SCZ-log_2Control >0) and downregulation in navy (log_2SCZ- log_2Control >0) of SCZ neuronal CRDs. Bottom plot shows a barplot of the number of up and downregulated SCZ neuronal CRDs per TRD from the ACC region. **(B)** Coefficients of SCZ heritability stratified by TRDs. The overlap of OCRs within the TRDs with SCZ risk variants was assessed using LD score regression. *P* values are from LD score regression. '#': significant for enrichment in LD score regression after Benjamini–Hochberg FDR correction for multiple testing across all tests in the plot (FDR < 5%). Error bars show standard error in SCZ heritability from LD score regression. **(C)** Top and bottom bar plot show $log_2FC_{fetal\_cortical}$ (Fetal cortical vs adult controls) and log_2FC (SCZ vs Controls) of neuronal ACC CRD respectively **(D)** Spearman correlation of $log_2FC_{fetal\_cortical}$ compared to log_2FC (SCZ vs Controls) of neuronal ACC CRDs from TRD1 (n=3,149 OCRs). P value is obtained from Spearman's rank correlation (ρ) test. **(E)** Functional pathway enrichment of OCRs from neuronal ACC TRD1 that overlap with fetal cortical specific OCRs.

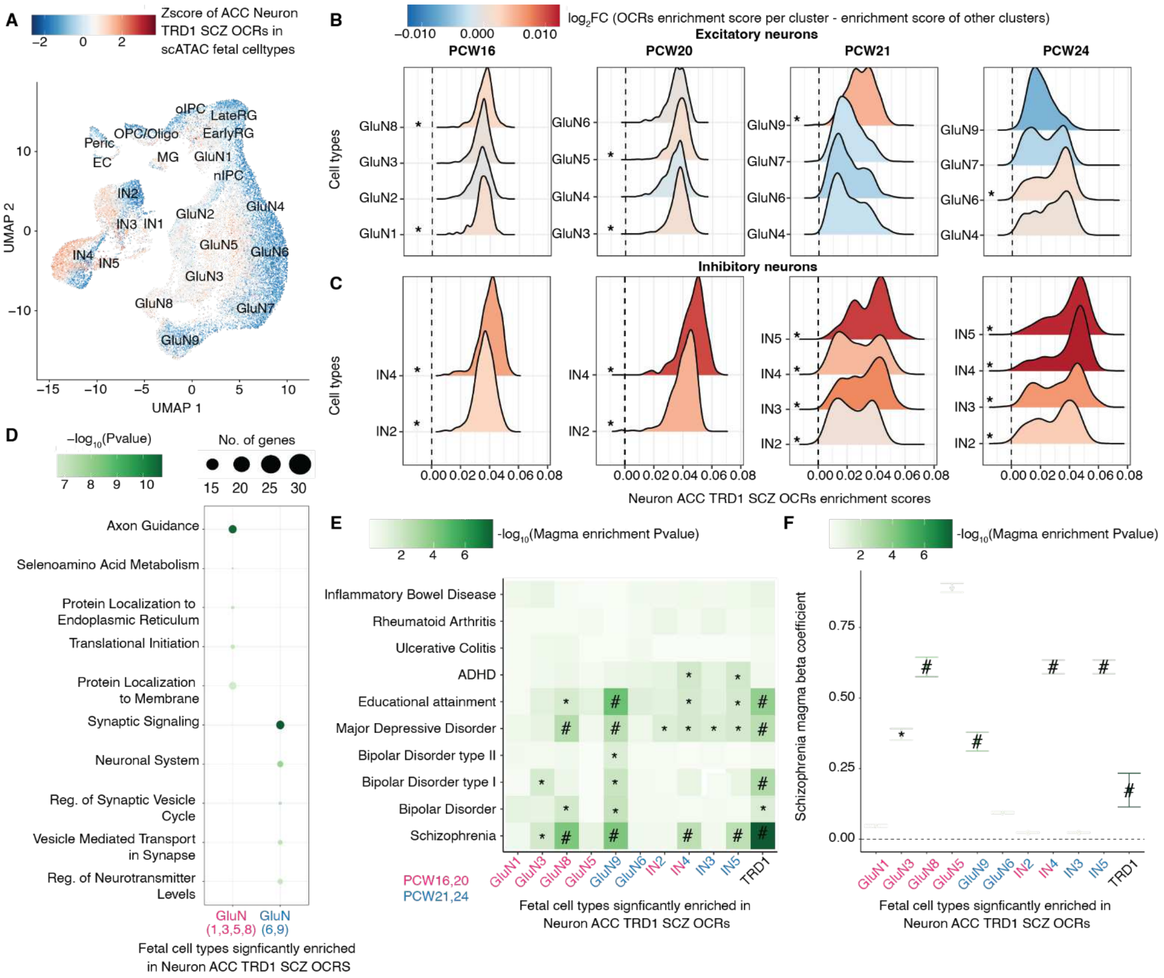

Figure S19 | Analysis of cell-type specificity of neuronal ACC schizophrenia OCRs in fetal cortical scATAC-seq data. A) UMAP plot of fetal cortical scATAC-seq data from Trevino *et al.* in which each cell type is colored by Z-scores of schizophrenia OCRs in neuronal ACC TRD1. The cell types include early radial glia (EarlyRG), late radial glia (LateRG), oligodendrocyte progenitor cell/oligodendrocyte (OPC/Oligo), neuronal intermediate progenitor cell (nIPC), oligo intermediate progenitor cell (oIPC), glutamatergic neuron (GluN), interneuron (IN), endothelial cell (EC), microglia (MG), and pericytes (Peric). **B**) Distribution of enrichment scores stratified by two major classes of cell types: 1) excitatory neurons (nine glutamatergic neurons) and 2) inhibitory neurons (five inhibitory neurons). The color represents the magnitude of coefficient of association of enrichment scores of schizophrenia OCRs from neuronal ACC TRD1 at each developmental stage separately for each cell types, using the model (enrichment score ~ celltype + (1| sample ID) where cell types are grouped as the cell type of interest vs. all other cell types. * depicts the cell types that are significantly enriched from the latter test and also have higher enrichment than the schizophrenia OCRs outside TRDs. **C**) Functional pathway analysis conducted on the early and late fetal glutamatergic specific marker genes that are also annotated by schizophrenia OCRs in TRD1. **D**) Heat map of MAGMA enrichment *P* values of brain-related and non-brain related GWAS traits and **E**) Coefficients of enrichment of common SCZ risk variants using MAGMA in fetal cell specific marker genes that are also annotated to schizophrenia OCRs within neuronal ACC TRD1. The x-axes in **D)** and **E)** are 1) early fetal (16+20 pcw) glutamatergic (GluN 1,3,5,8) in magenta, 2) late fetal (21+24 pcw) glutamatergic (GluN 6,9) in blue, 3) early fetal (16+20 pcw) inhibitory (IN2,4) in magenta, 4) late fetal (21+24 pcw) inhibitory (IN 2,3,4,5) in blue fetal cell types and all ABC mapped schizophrenia OCRs to genes within neuronal ACC TRD1 in black. ‘#’:

significant MAGMA enrichment coefficient after FDR correction of multiple testing across all tests in the plot (Benjamini–Hochberg test); ‘*’: nominally significant for enrichment in (**D**) and (**E**).

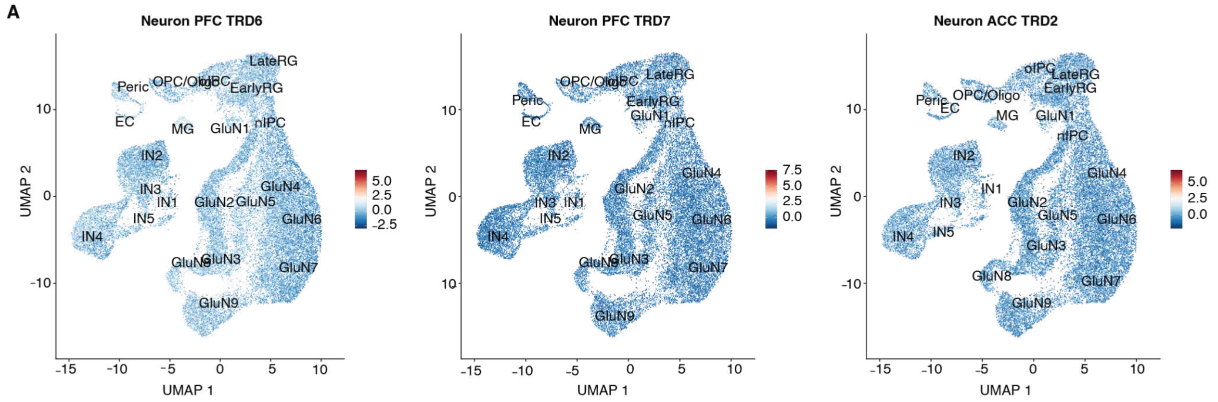

Figure S20 | Enrichment of expression of schizophrenia OCRs in SCZ TRDs in fetal cortical OCRs in scATAC-seq data. A) UMAP plot of fetal cortical scATAC-seq data from Trevino *et al.* in which each cell type is colored by Z-scores of neuronal PFC schizophrenia OCRs in neuronal PFC TRD6, neuronal PFC TRD7 and neuronal ACC schizophrenia OCRs in neuronal ACC TRD2. The cell types include early radial glia (EarlyRG), late radial glia (LateRG), oligodendrocyte progenitor cell/oligodendrocyte (OPC/Oligo), neuronal intermediate progenitor cell (nIPC), oligo intermediate progenitor cell (oIPC), glutamatergic neuron (GluN), interneuron (IN), endothelial cell (EC), microglia (MG), and pericytes (Peric).

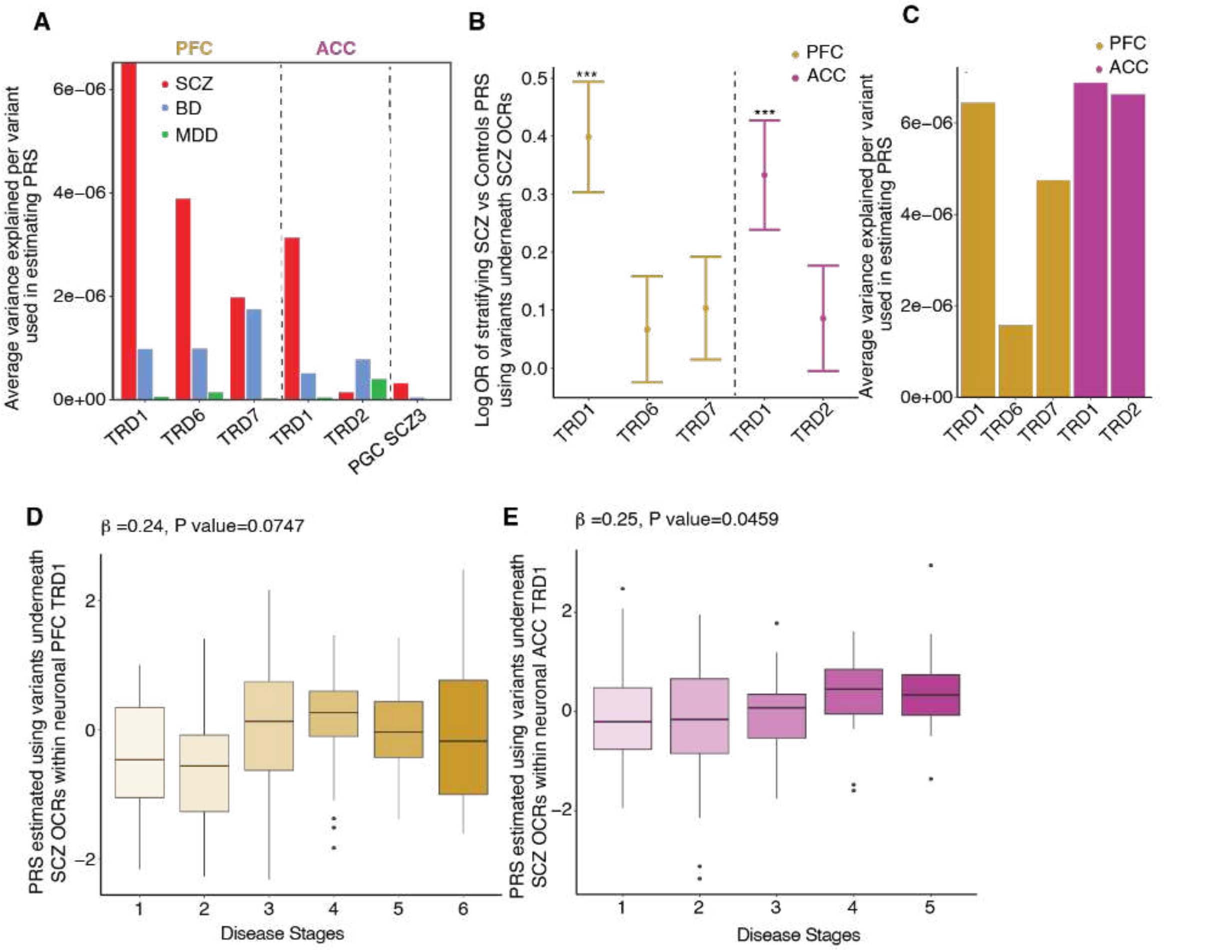

Figure S21 | Trans Regulatory Domain one (TRD1) in PFC and ACC can stratify SCZ cases and controls in MVP and CMC cohort. For SCZ TRDs: TRD1, TRD6 and TRD7 from PFC region and TRD1, TRD2 from ACC region. **A**) Average variance explained per variant used in estimating PRS of MVP cohort (PFC TRDs: 1,388 for TRD1, 139 for TRD6 and 103 for TRD7; ACC TRDs: 815 for TRD1 and 46 for TRD2; PGC3 SCZ: 350,882). **B**) Plot of logOR from logistic regression of PRS of 632 individuals from CMC cohort estimated using the SNPs in OCRs from these TRDs to predict their case and control status, **C**) Average variance explained per variant used in estimating PRS of CMC cohort (PFC TRDs: 3,869 for TRD1, 449 for TRD6 and 357 for TRD7; ACC TRDs: 2,447 for TRD1 and 172 for TRD2; PGC3 SCZ: 350,882). **D-E)** Turkey bar plot of PRS of samples calculated using the SNPs underneath schizophrenia OCRs within TRD1 (subsetted from the full PGC3 SCZ GWAS summary statistics), stratified by inferred disease severity stages in PFC and ACC respectively.

Supplementary Tables

Table S1: Metadata and summary of QC metrics for neuronal PFC, neuronal ACC, non-neuronal PFC and non-neuronal ACC samples.

Table S2: GWAS enrichment of brain traits and non-brain related traits in neuronal and non-neuronal OCRs.

Table S3: Inferred stages of SCZ/controls of neuronal and non-neuronal samples in PFC and ACC regions using manifold learning method and their respective polygenic risk scores estimated using PGC3 SCZ GWAS summary statistics.

Table S4: List of genomic coordinates of identified neuronal PFC, neuronal ACC, non-neuronal PFC and non-neuronal ACC CRDs.

Table S5: GWAS enrichment of brain traits and non-brain related traits in OCRs within neuron PFC, neuron ACC, non_neuron PFC and non_neuron ACC CRDs stratified by OCR location (inside or outside CRDs).

Table S6: GWAS enrichment of brain traits and non-brain related traits of schizophrenia OCRs that reside inside and outside of neuronal PFC, neuronal ACC, non-neuronal PFC and non-neuronal ACC CRDs.

Table S7: Association test of scFetal cell types with neuronal TRD expression scores in PFC and ACC regions. Magma enrichment of brain and non-brain related traits in significantly associated scfetal cell types from association test with TRD1 marker genes that are present within the neuron TRD1 in PFC and ACC regions. List of TRD1 associated scfetal cell types marker genes and their functional pathways analysis using GREAT tool.

Supplementary references

1. J. D. Buenrostro, B. Wu, H. Y. Chang, W. J. Greenleaf, ATAC-seq: A method for assaying chromatin accessibility genome-wide. *Curr. Protoc. Mol. Biol.* **109**, 21.29.1–21.29.9 (2015).

2. J. Bendl, M. E. Hauberg, K. Girdhar, E. Im, J. M. Vicari, S. Rahman, M. B. Fernando, K. G. Townsley, P. Dong, R. Misir, S. P. Kleopoulos, S. M. Reach, P. Apontes, B. Zeng, W. Zhang, G. Voloudakis, K. J. Brennand, R. A. Nixon, V. Haroutunian, G. E. Hoffman, J. F. Fullard, P. Roussos, The three-dimensional landscape of cortical chromatin accessibility in Alzheimer's disease. *Nat. Neurosci.* **25**, 1366–1378 (2022).

3. M. E. Hauberg, J. Creus-Muncunill, J. Bendl, A. Kozlenkov, B. Zeng, C. Corwin, S. Chowdhury, H. Kranz, Y. L. Hurd, M. Wegner, A. D. Børglum, S. Dracheva, M. E. Ehrlich, J. F. Fullard, P. Roussos, Common schizophrenia risk variants are enriched in open chromatin regions of human glutamatergic neurons. *Nat. Commun.* **11**, 5581 (2020).

4. A. M. Bolger, M. Lohse, B. Usadel, Trimmomatic: a flexible trimmer for Illumina sequence data. *Bioinformatics*. **30**, 2114–2120 (2014).

5. A. Dobin, C. A. Davis, F. Schlesinger, J. Drenkow, C. Zaleski, S. Jha, P. Batut, M. Chaisson, T. R. Gingeras, STAR: ultrafast universal RNA-seq aligner. *Bioinformatics*. **29**, 15–21 (2013).

6. B. van de Geijn, G. McVicker, Y. Gilad, J. K. Pritchard, WASP: allele-specific software for robust molecular quantitative trait locus discovery. *Nat. Methods*. **12**, 1061–1063 (2015).

7. H. Li, B. Handsaker, A. Wysoker, T. Fennell, J. Ruan, N. Homer, G. Marth, G. Abecasis, R. Durbin, 1000 Genome Project Data Processing Subgroup, The Sequence Alignment/Map format and SAMtools. *Bioinformatics*. **25**, 2078–2079 (2009).

8. S. G. Landt, G. K. Marinov, A. Kundaje, P. Kheradpour, F. Pauli, S. Batzoglou, B. E. Bernstein, P. Bickel, J. B. Brown, P. Cayting, Y. Chen, G. DeSalvo, C. Epstein, K. I. Fisher-Aylor, G. Euskirchen, M. Gerstein, J. Gertz, A. J. Hartemink, M. M. Hoffman, V. R. Iyer, Y. L. Jung, S. Karmakar, M. Kellis, P. V. Kharchenko, Q. Li, T. Liu, X. S. Liu, L. Ma, A. Milosavljevic, R. M. Myers, P. J. Park, M. J. Pazin, M. D. Perry, D. Raha, T. E. Reddy, J. Rozowsky, N. Shoresh, A. Sidow, M. Slattery, J. A. Stamatoyannopoulos, M. Y. Tolstorukov, K. P. White, S. Xi, P. J. Farnham, J. D. Lieb, B. J. Wold, M. Snyder, ChIP-seq guidelines and practices of the ENCODE and modENCODE consortia. *Genome Res.* **22**, 1813–1831 (2012).

9. P. Orchard, Y. Kyono, J. Hensley, J. O. Kitzman, S. C. J. Parker, Quantification, Dynamic Visualization, and Validation of Bias in ATAC-Seq Data with ataqv. *Cell Syst*. **10**, 298–306.e4 (2020).

10. A. McKenna, M. Hanna, E. Banks, A. Sivachenko, K. Cibulskis, A. Kernytsky, K. Garimella, D. Altshuler, S. Gabriel, M. Daly, M. A. DePristo, The Genome Analysis Toolkit: a MapReduce framework for analyzing next-generation DNA sequencing data. *Genome Res.* **20**, 1297–1303 (2010).

11. H. M. Amemiya, A. Kundaje, A. P. Boyle, The ENCODE Blacklist: Identification of Problematic Regions of the Genome. *Sci. Rep.* **9**, 9354 (2019).

12. S. T. Sherry, M. H. Ward, M. Kholodov, J. Baker, L. Phan, E. M. Smigielski, K. Sirotkin, dbSNP: the NCBI database of genetic variation. *Nucleic Acids Res.* **29**, 308–311 (2001).

13. A. Manichaikul, J. C. Mychaleckyj, S. S. Rich, K. Daly, M. Sale, W.-M. Chen, Robust relationship inference in genome-wide association studies. *Bioinformatics*. **26**, 2867–2873

(2010).

14. C. P. Fulco, J. Nasser, T. R. Jones, G. Munson, D. T. Bergman, V. Subramanian, S. R. Grossman, R. Anyoha, B. R. Doughty, T. A. Patwardhan, T. H. Nguyen, M. Kane, E. M. Perez, N. C. Durand, C. A. Lareau, E. K. Stamenova, E. L. Aiden, E. S. Lander, J. M. Engreitz, Activity-by-contact model of enhancer-promoter regulation from thousands of CRISPR perturbations. *Nat. Genet.* **51**, 1664–1669 (2019).

15. P. Dong, G. E. Hoffman, P. Apontes, J. Bendl, S. Rahman, M. B. Fernando, B. Zeng, J. M. Vicari, W. Zhang, K. Girdhar, K. G. Townsley, R. Misir, CommonMind Consortium, K. J. Brennand, V. Haroutunian, G. Voloudakis, J. F. Fullard, P. Roussos, Population-level variation in enhancer expression identifies disease mechanisms in the human brain. *Nat. Genet.* **54**, 1493–1503 (2022).

16. M. D. Robinson, D. J. McCarthy, G. K. Smyth, edgeR: a Bioconductor package for differential expression analysis of digital gene expression data. *Bioinformatics*. **26**, 139–140 (2010).

17. G. E. Hoffman, E. E. Schadt, variancePartition: interpreting drivers of variation in complex gene expression studies. *BMC Bioinformatics*. **17**, 483 (2016).

18. G. E. Hoffman, P. Roussos, Dream: powerful differential expression analysis for repeated measures designs. *Bioinformatics*. **37**, 192–201 (2021).

19. G. E. Hoffman, J. Bendl, G. Voloudakis, K. S. Montgomery, L. Sloofman, Y.-C. Wang, H. R. Shah, M. E. Hauberg, J. S. Johnson, K. Girdhar, L. Song, J. F. Fullard, R. Kramer, C.-G. Hahn, R. Gur, S. Marenco, B. K. Lipska, D. A. Lewis, V. Haroutunian, S. Hemby, P. Sullivan, S. Akbarian, A. Chess, J. D. Buxbaum, G. E. Crawford, E. Domenici, B. Devlin, S. K. Sieberts, M. A. Peters, P. Roussos, CommonMind Consortium provides transcriptomic and epigenomic data for Schizophrenia and Bipolar Disorder. *Sci Data*. **6**, 180 (2019).

20. R. E. Kass, A. E. Raftery, Bayes Factors. *J. Am. Stat. Assoc.* **90**, 773–795 (1995).

21. L. McInnes, J. Healy, J. Melville, UMAP: Uniform Manifold Approximation and Projection for Dimension Reduction. *arXiv [stat.ML]* (2018), (available at http://arxiv.org/abs/1802.03426).

22. R. R. Coifman, S. Lafon, Diffusion maps. *Appl. Comput. Harmon. Anal.* **21**, 5–30 (2006).

23. L. Haghverdi, M. Büttner, F. A. Wolf, F. Buettner, F. J. Theis, Diffusion pseudotime robustly reconstructs lineage branching. *Nat. Methods*. **13**, 845–848 (2016).

24. V. A. Traag, L. Waltman, N. J. van Eck, From Louvain to Leiden: guaranteeing well-connected communities. *Sci. Rep.* **9**, 5233 (2019).

25. J. M. Gaziano, J. Concato, M. Brophy, L. Fiore, S. Pyarajan, J. Breeling, S. Whitbourne, J. Deen, C. Shannon, D. Humphries, P. Guarino, M. Aslan, D. Anderson, R. LaFleur, T. Hammond, K. Schaa, J. Moser, G. Huang, S. Muralidhar, R. Przygodzki, T. J. O'Leary, Million Veteran Program: A mega-biobank to study genetic influences on health and disease. *J. Clin. Epidemiol.* **70**, 214–223 (2016).

26. K. M. Harrington, X.-M. T. Nguyen, R. J. Song, K. Hannagan, R. Quaden, D. R. Gagnon, K. Cho, J. E. Deen, S. Muralidhar, T. J. O'Leary, J. M. Gaziano, S. B. Whitbourne, VA Million Veteran Program, Gender Differences in Demographic and Health Characteristics of the Million Veteran Program Cohort. *Womens. Health Issues*. **29 Suppl 1**, S56–S66 (2019).

27. J. Gelernter, N. Sun, R. Polimanti, R. Pietrzak, D. F. Levey, J. Bryois, Q. Lu, Y. Hu, B. Li, K. Radhakrishnan, M. Aslan, K.-H. Cheung, Y. Li, N. Rajeevan, F. Sayward, K. Harrington, Q.

Chen, K. Cho, S. Pyarajan, P. F. Sullivan, R. Quaden, Y. Shi, H. Hunter-Zinck, J. M. Gaziano, J. Concato, H. Zhao, M. B. Stein, Department of Veterans Affairs Cooperative Studies Program (#575B) and Million Veteran Program, Genome-wide association study of post-traumatic stress disorder reexperiencing symptoms in >165,000 US veterans. *Nat. Neurosci.* **22**, 1394–1401 (2019).

28. A. T. Marees, H. de Kluiver, S. Stringer, F. Vorspan, E. Curis, C. Marie-Claire, E. M. Derks, A tutorial on conducting genome-wide association studies: Quality control and statistical analysis. *Int. J. Methods Psychiatr. Res.* **27**, e1608 (2018).

29. H. Fang, Q. Hui, J. Lynch, J. Honerlaw, T. L. Assimes, J. Huang, M. Vujkovic, S. M. Damrauer, S. Pyarajan, J. M. Gaziano, S. L. DuVall, C. J. O'Donnell, K. Cho, K.-M. Chang, P. W. F. Wilson, P. S. Tsao, VA Million Veteran Program, Y. V. Sun, H. Tang, Harmonizing Genetic Ancestry and Self-identified Race/Ethnicity in Genome-wide Association Studies. *Am. J. Hum. Genet.* **105**, 763–772 (2019).

30. 1000 Genomes Project Consortium, G. R. Abecasis, A. Auton, L. D. Brooks, M. A. DePristo, R. M. Durbin, R. E. Handsaker, H. M. Kang, G. T. Marth, G. A. McVean, An integrated map of genetic variation from 1,092 human genomes. *Nature*. **491**, 56–65 (2012).

31. S. K. Sieberts, T. M. Perumal, M. M. Carrasquillo, M. Allen, J. S. Reddy, G. E. Hoffman, K. K. Dang, J. Calley, P. J. Ebert, J. Eddy, X. Wang, A. K. Greenwood, S. Mostafavi, CommonMind Consortium (CMC), The AMP-AD Consortium, L. Omberg, M. A. Peters, B. A. Logsdon, P. L. De Jager, N. Ertekin-Taner, L. M. Mangravite, Large eQTL meta-analysis reveals differing patterns between cerebral cortical and cerebellar brain regions. *Sci Data*. **7**, 340 (2020).

32. D. Taliun, D. N. Harris, M. D. Kessler, J. Carlson, Z. A. Szpiech, R. Torres, S. A. G. Taliun, A. Corvelo, S. M. Gogarten, H. M. Kang, A. N. Pitsillides, J. LeFaive, S.-B. Lee, X. Tian, B. L. Browning, S. Das, A.-K. Emde, W. E. Clarke, D. P. Loesch, A. C. Shetty, T. W. Blackwell, A. V. Smith, Q. Wong, X. Liu, M. P. Conomos, D. M. Bobo, F. Aguet, C. Albert, A. Alonso, K. G. Ardlie, D. E. Arking, S. Aslibekyan, P. L. Auer, J. Barnard, R. G. Barr, L. Barwick, L. C. Becker, R. L. Beer, E. J. Benjamin, L. F. Bielak, J. Blangero, M. Boehnke, D. W. Bowden, J. A. Brody, E. G. Burchard, B. E. Cade, J. F. Casella, B. Chalazan, D. I. Chasman, Y.-D. I. Chen, M. H. Cho, S. H. Choi, M. K. Chung, C. B. Clish, A. Correa, J. E. Curran, B. Custer, D. Darbar, M. Daya, M. de Andrade, D. L. DeMeo, S. K. Dutcher, P. T. Ellinor, L. S. Emery, C. Eng, D. Fatkin, T. Fingerlin, L. Forer, M. Fornage, N. Franceschini, C. Fuchsberger, S. M. Fullerton, S. Germer, M. T. Gladwin, D. J. Gottlieb, X. Guo, M. E. Hall, J. He, N. L. Heard-Costa, S. R. Heckbert, M. R. Irvin, J. M. Johnsen, A. D. Johnson, R. Kaplan, S. L. R. Kardia, T. Kelly, S. Kelly, E. E. Kenny, D. P. Kiel, R. Klemmer, B. A. Konkle, C. Kooperberg, A. Köttgen, L. A. Lange, J. Lasky-Su, D. Levy, X. Lin, K.-H. Lin, C. Liu, R. J. F. Loos, L. Garman, R. Gerszten, S. A. Lubitz, K. L. Lunetta, A. C. Y. Mak, A. Manichaikul, A. K. Manning, R. A. Mathias, D. D. McManus, S. T. McGarvey, J. B. Meigs, D. A. Meyers, J. L. Mikulla, M. A. Minear, B. D. Mitchell, S. Mohanty, M. E. Montasser, C. Montgomery, A. C. Morrison, J. M. Murabito, A. Natale, P. Natarajan, S. C. Nelson, K. E. North, J. R. O'Connell, N. D. Palmer, N. Pankratz, G. M. Peloso, P. A. Peyser, J. Pleiness, W. S. Post, B. M. Psaty, D. C. Rao, S. Redline, A. P. Reiner, D. Roden, J. I. Rotter, I. Ruczinski, C. Sarnowski, S. Schoenherr, D. A. Schwartz, J.-S. Seo, S. Seshadri, V. A. Sheehan, W. H. Sheu, M. B. Shoemaker, N. L. Smith, J. A. Smith, N. Sotoodehnia, A. M. Stilp, W. Tang, K. D. Taylor, M. Telen, T. A. Thornton, R. P. Tracy, D. J. Van Den Berg, R. S. Vasan, K. A. Viaud-Martinez, S. Vrieze, D. E. Weeks, B. S. Weir, S. T. Weiss, L.-C. Weng, C. J. Willer, Y. Zhang, X. Zhao, D. K. Arnett, A. E. Ashley-Koch, K. C. Barnes, E. Boerwinkle, S. Gabriel, R. Gibbs, K. M. Rice, S. S. Rich, E. K. Silverman, P. Qasba, W. Gan, NHLBI Trans-Omics for Precision Medicine (TOPMed) Consortium, G. J. Papanicolaou, D. A. Nickerson, S. R. Browning, M. C. Zody, S. Zöllner, J. G. Wilson, L. A. Cupples, C. C. Laurie, C. E. Jaquish, R. D. Hernandez, T. D. O'Connor, G. R. Abecasis, Sequencing of 53,831 diverse genomes from the NHLBI TOPMed Program. *Nature*. **590**, 290–

299 (2021).

33. 1000 Genomes Project Consortium, A. Auton, L. D. Brooks, R. M. Durbin, E. P. Garrison, H. M. Kang, J. O. Korbel, J. L. Marchini, S. McCarthy, G. A. McVean, G. R. Abecasis, A global reference for human genetic variation. *Nature*. **526**, 68–74 (2015).

34. P. Danecek, J. K. Bonfield, J. Liddle, J. Marshall, V. Ohan, M. O. Pollard, A. Whitwham, T. Keane, S. A. McCarthy, R. M. Davies, H. Li, Twelve years of SAMtools and BCFtools. *Gigascience*. **10** (2021), doi:10.1093/gigascience/giab008.

35. C. C. Chang, C. C. Chow, L. C. Tellier, S. Vattikuti, S. M. Purcell, J. J. Lee, Second-generation PLINK: rising to the challenge of larger and richer datasets. *Gigascience*. **4**, 7 (2015).

36. V. Trubetskoy, A. F. Pardiñas, T. Qi, G. Panagiotaropoulou, S. Awasthi, T. B. Bigdeli, J. Bryois, C.-Y. Chen, C. A. Dennison, L. S. Hall, M. Lam, K. Watanabe, O. Frei, T. Ge, J. C. Harwood, F. Koopmans, S. Magnusson, A. L. Richards, J. Sidorenko, Y. Wu, J. Zeng, J. Grove, M. Kim, Z. Li, G. Voloudakis, W. Zhang, M. Adams, I. Agartz, E. G. Atkinson, E. Agerbo, M. Al Eissa, M. Albus, M. Alexander, B. Z. Alizadeh, K. Alptekin, T. D. Als, F. Amin, V. Arolt, M. Arrojo, L. Athanasiu, M. H. Azevedo, S. A. Bacanu, N. J. Bass, M. Begemann, R. A. Belliveau, J. Bene, B. Benyamin, S. E. Bergen, G. Blasi, J. Bobes, S. Bonassi, A. Braun, R. A. Bressan, E. J. Bromet, R. Bruggeman, P. F. Buckley, R. L. Buckner, J. Bybjerg-Grauholm, W. Cahn, M. J. Cairns, M. E. Calkins, V. J. Carr, D. Castle, S. V. Catts, K. D. Chambert, R. C. K. Chan, B. Chaumette, W. Cheng, E. F. C. Cheung, S. A. Chong, D. Cohen, A. Consoli, Q. Cordeiro, J. Costas, C. Curtis, M. Davidson, K. L. Davis, L. de Haan, F. Degenhardt, L. E. DeLisi, D. Demontis, F. Dickerson, D. Dikeos, T. Dinan, S. Djurovic, J. Duan, G. Ducci, F. Dudbridge, J. G. Eriksson, L. Fañanás, S. V. Faraone, A. Fiorentino, A. Forstner, J. Frank, N. B. Freimer, M. Fromer, A. Frustaci, A. Gadelha, G. Genovese, E. S. Gershon, M. Giannitelli, I. Giegling, P. Giusti-Rodríguez, S. Godard, J. I. Goldstein, J. González Peñas, A. González-Pinto, S. Gopal, J. Gratten, M. F. Green, T. A. Greenwood, O. Guillin, S. Gülöksüz, R. E. Gur, R. C. Gur, B. Gutiérrez, E. Hahn, H. Hakonarson, V. Haroutunian, A. M. Hartmann, C. Harvey, C. Hayward, F. A. Henskens, S. Herms, P. Hoffmann, D. P. Howrigan, M. Ikeda, C. Iyegbe, I. Joa, A. Julià, A. K. Kähler, T. Kam-Thong, Y. Kamatani, S. Karachanak-Yankova, O. Kebir, M. C. Keller, B. J. Kelly, A. Khrunin, S.-W. Kim, J. Klovins, N. Kondratiev, B. Konte, J. Kraft, M. Kubo, V. Kučinskas, Z. A. Kučinskiene, A. Kusumawardhani, H. Kuzelova-Ptackova, S. Landi, L. C. Lazzeroni, P. H. Lee, S. E. Legge, D. S. Lehrer, R. Lencer, B. Lerer, M. Li, J. Lieberman, G. A. Light, S. Limborska, C.-M. Liu, J. Lönnqvist, C. M. Loughland, J. Lubinski, J. J. Luykx, A. Lynham, M. Macek Jr, A. Mackinnon, P. K. E. Magnusson, B. S. Maher, W. Maier, D. Malaspina, J. Mallet, S. R. Marder, S. Marsal, A. R. Martin, L. Martorell, M. Mattheisen, R. W. McCarley, C. McDonald, J. J. McGrath, H. Medeiros, S. Meier, B. Melegh, I. Melle, R. I. Mesholam-Gately, A. Metspalu, P. T. Michie, L. Milani, V. Milanova, M. Mitjans, E. Molden, E. Molina, M. D. Molto, V. Mondelli, C. Moreno, C. P. Morley, G. Muntané, K. C. Murphy, I. Myin-Germeys, I. Nenadić, G. Nestadt, L. Nikitina-Zake, C. Noto, K. H. Nuechterlein, N. L. O'Brien, F. A. O'Neill, S.-Y. Oh, A. Olincy, V. K. Ota, C. Pantelis, G. N. Papadimitriou, M. Parellada, T. Paunio, R. Pellegrino, S. Periyasamy, D. O. Perkins, B. Pfuhlmann, O. Pietiläinen, J. Pimm, D. Porteous, J. Powell, D. Quattrone, D. Quested, A. D. Radant, A. Rampino, M. H. Rapaport, A. Rautanen, A. Reichenberg, C. Roe, J. L. Roffman, J. Roth, M. Rothermundt, B. P. F. Rutten, S. Saker-Delye, V. Salomaa, J. Sanjuan, M. L. Santoro, A. Savitz, U. Schall, R. J. Scott, L. J. Seidman, S. I. Sharp, J. Shi, L. J. Siever, E. Sigurdsson, K. Sim, N. Skarabis, P. Slominsky, H.-C. So, J. L. Sobell, E. Söderman, H. J. Stain, N. E. Steen, A. A. Steixner-Kumar, E. Stögmann, W. S. Stone, R. E. Straub, F. Streit, E. Strengman, T. S. Stroup, M. Subramaniam, C. A. Sugar, J. Suvisaari, D. M. Svrakic, N. R. Swerdlow, J. P. Szatkiewicz, T. M. T. Ta, A. Takahashi, C. Terao, F. Thibaut, D. Toncheva, P. A. Tooney, S. Torretta, S. Tosato, G. B. Tura, B. I. Turetsky, A. Üçok, A. Vaaler, T. van Amelsvoort, R. van Winkel, J. Veijola, J. Waddington, H. Walter, A. Waterreus, B. T. Webb, M. Weiser, N. M. Williams, S. H. Witt, B. K. Wormley, J. Q. Wu, Z. Xu, R. Yolken, C. C. Zai, W. Zhou, F. Zhu, F. Zimprich, E. C. Atbaşoğlu, M. Ayub, C. Benner,

A. Bertolino, D. W. Black, N. J. Bray, G. Breen, N. G. Buccola, W. F. Byerley, W. J. Chen, C. R. Cloninger, B. Crespo-Facorro, G. Donohoe, R. Freedman, C. Galletly, M. J. Gandal, M. Gennarelli, D. M. Hougaard, H.-G. Hwu, A. V. Jablensky, S. A. McCarroll, J. L. Moran, O. Mors, P. B. Mortensen, B. Müller-Myhsok, A. L. Neil, M. Nordentoft, M. T. Pato, T. L. Petryshen, M. Pirinen, A. E. Pulver, T. G. Schulze, J. M. Silverman, J. W. Smoller, E. A. Stahl, D. W. Tsuang, E. Vilella, S.-H. Wang, S. Xu, Indonesia Schizophrenia Consortium, PsychENCODE, Psychosis Endophenotypes International Consortium, SynGO Consortium, R. Adolfsson, C. Arango, B. T. Baune, S. I. Belangero, A. D. Børglum, D. Braff, E. Bramon, J. D. Buxbaum, D. Campion, J. A. Cervilla, S. Cichon, D. A. Collier, A. Corvin, D. Curtis, M. D. Forti, E. Domenici, H. Ehrenreich, V. Escott-Price, T. Esko, A. H. Fanous, A. Gareeva, M. Gawlik, P. V. Gejman, M. Gill, S. J. Glatt, V. Golimbet, K. S. Hong, C. M. Hultman, S. E. Hyman, N. Iwata, E. G. Jönsson, R. S. Kahn, J. L. Kennedy, E. Khusnutdinova, G. Kirov, J. A. Knowles, M.-O. Krebs, C. Laurent-Levinson, J. Lee, T. Lencz, D. F. Levinson, Q. S. Li, J. Liu, A. K. Malhotra, D. Malhotra, A. McIntosh, A. McQuillin, P. R. Menezes, V. A. Morgan, D. W. Morris, B. J. Mowry, R. M. Murray, V. Nimgaonkar, M. M. Nöthen, R. A. Ophoff, S. A. Paciga, A. Palotie, C. N. Pato, S. Qin, M. Rietschel, B. P. Riley, M. Rivera, D. Rujescu, M. C. Saka, A. R. Sanders, S. G. Schwab, A. Serretti, P. C. Sham, Y. Shi, D. St Clair, H. Stefánsson, K. Stefansson, M. T. Tsuang, J. van Os, M. P. Vawter, D. R. Weinberger, T. Werge, D. B. Wildenauer, X. Yu, W. Yue, P. A. Holmans, A. J. Pocklington, P. Roussos, E. Vassos, M. Verhage, P. M. Visscher, J. Yang, D. Posthuma, O. A. Andreassen, K. S. Kendler, M. J. Owen, N. R. Wray, M. J. Daly, H. Huang, B. M. Neale, P. F. Sullivan, S. Ripke, J. T. R. Walters, M. C. O'Donovan, Schizophrenia Working Group of the Psychiatric Genomics Consortium, Mapping genomic loci implicates genes and synaptic biology in schizophrenia. *Nature*. **604**, 502–508 (2022).

37. T. Ge, C.-Y. Chen, Y. Ni, Y.-C. A. Feng, J. W. Smoller, Polygenic prediction via Bayesian regression and continuous shrinkage priors. *Nat. Commun.* **10**, 1776 (2019).

38. C. Sudlow, J. Gallacher, N. Allen, V. Beral, P. Burton, J. Danesh, P. Downey, P. Elliott, J. Green, M. Landray, B. Liu, P. Matthews, G. Ong, J. Pell, A. Silman, A. Young, T. Sprosen, T. Peakman, R. Collins, UK biobank: an open access resource for identifying the causes of a wide range of complex diseases of middle and old age. *PLoS Med.* **12**, e1001779 (2015).

39. K. Girdhar, G. E. Hoffman, J. Bendl, S. Rahman, P. Dong, W. Liao, M. E. Hauberg, L. Sloofman, L. Brown, O. Devillers, B. S. Kassim, J. R. Wiseman, R. Park, E. Zharovsky, R. Jacobov, E. Flatow, A. Kozlenkov, T. Gilgenast, J. S. Johnson, L. Couto, M. A. Peters, J. E. Phillips-Cremins, C.-G. Hahn, R. E. Gur, C. A. Tamminga, D. A. Lewis, V. Haroutunian, PsychENCODE Consortium, S. Dracheva, B. K. Lipska, S. Marenco, M. Kundakovic, J. F. Fullard, Y. Jiang, P. Roussos, S. Akbarian, Chromatin domain alterations linked to 3D genome organization in a large cohort of schizophrenia and bipolar disorder brains. *Nat. Neurosci.* **25**, 474–483 (2022).

40. O. Stegle, L. Parts, M. Piipari, J. Winn, R. Durbin, Using probabilistic estimation of expression residuals (PEER) to obtain increased power and interpretability of gene expression analyses. *Nat. Protoc.* **7**, 500–507 (2012).

41. G. E. Hoffman, J. Bendl, K. Girdhar, P. Roussos, decorate: differential epigenetic correlation test. *Bioinformatics*. **36**, 2856–2861 (2020).

42. R. A. Fisher, "Statistical Methods for Research Workers" in *Breakthroughs in Statistics: Methodology and Distribution*, S. Kotz, N. L. Johnson, Eds. (Springer New York, New York, NY, 1992), pp. 66–70.

43. ENCODE Project Consortium, An integrated encyclopedia of DNA elements in the human genome. *Nature*. **489**, 57–74 (2012).

44. K. Van den Berge, C. Soneson, M. D. Robinson, L. Clement, stageR: a general stage-wise method for controlling the gene-level false discovery rate in differential expression and differential transcript usage. *Genome Biol.* **18**, 151 (2017).

45. R. E. Blakesley, S. Mazumdar, M. A. Dew, P. R. Houck, G. Tang, C. F. Reynolds 3rd, M. A. Butters, Comparisons of methods for multiple hypothesis testing in neuropsychological research. *Neuropsychology*. **23**, 255–264 (2009).

46. D. S. Chiu, A. Talhouk, diceR: an R package for class discovery using an ensemble driven approach. *BMC Bioinformatics*. **19**, 11 (2018).

47. F. Murtagh, P. Contreras, Algorithms for hierarchical clustering: an overview. *Wiley Interdiscip. Rev. Data Min. Knowl. Discov.* **2**, 86–97 (2012).

48. F. B. Baker, L. J. Hubert, Measuring the Power of Hierarchical Cluster Analysis. *J. Am. Stat. Assoc.* **70**, 31–38 (1975).

49. S. Rahman, P. Dong, P. Apontes, M. B. Fernando, K. G. Townsley, K. Girdhar, J. Bendl, Z. Shao, R. Misir, N. Tsankova, S. P. Kleopoulos, K. J. Brennand, J. F. Fullard, P. Roussos, From compartments to gene loops: Functions of the 3D genome in the human brain. *bioRxiv* (2021), p. 2021.10.12.464094.

50. A. E. Trevino, F. Müller, J. Andersen, L. Sundaram, A. Kathiria, A. Shcherbina, K. Farh, H. Y. Chang, A. M. Pașca, A. Kundaje, S. P. Pașca, W. J. Greenleaf, Chromatin and gene-regulatory dynamics of the developing human cerebral cortex at single-cell resolution. *Cell*. **184**, 5053–5069.e23 (2021).

51. Y. Hao, T. Stuart, M. Kowalski, S. Choudhary, P. Hoffman, A. Hartman, A. Srivastava, G. Molla, S. Madad, C. Fernandez-Granda, R. Satija, Dictionary learning for integrative, multimodal, and scalable single-cell analysis. *bioRxiv* (2022), p. 2022.02.24.481684.

52. C. A. de Leeuw, J. M. Mooij, T. Heskes, D. Posthuma, MAGMA: generalized gene-set analysis of GWAS data. *PLoS Comput. Biol.* **11**, e1004219 (2015).

53. W. Zhang, G. Voloudakis, V. M. Rajagopal, B. Readhead, J. T. Dudley, E. E. Schadt, J. L. M. Björkegren, Y. Kim, J. F. Fullard, G. E. Hoffman, P. Roussos, Integrative transcriptome imputation reveals tissue-specific and shared biological mechanisms mediating susceptibility to complex traits. *Nat. Commun.* **10**, 3834 (2019).

54. A. Zeisel, A. B. Muñoz-Manchado, S. Codeluppi, P. Lönnerberg, G. La Manno, A. Juréus, S. Marques, H. Munguba, L. He, C. Betsholtz, C. Rolny, G. Castelo-Branco, J. Hjerling-Leffler, S. Linnarsson, Brain structure. Cell types in the mouse cortex and hippocampus revealed by single-cell RNA-seq. *Science*. **347**, 1138–1142 (2015).

55. J. Bryois, M. E. Garrett, L. Song, A. Safi, P. Giusti-Rodriguez, G. D. Johnson, A. W. Shieh, A. Buil, J. F. Fullard, P. Roussos, P. Sklar, S. Akbarian, V. Haroutunian, C. A. Stockmeier, G. A. Wray, K. P. White, C. Liu, T. E. Reddy, A. Ashley-Koch, P. F. Sullivan, G. E. Crawford, Evaluation of chromatin accessibility in prefrontal cortex of individuals with schizophrenia. *Nat. Commun.* **9**, 3121 (2018).

56. J. F. Fullard, M. E. Hauberg, J. Bendl, G. Egervari, M.-D. Cirnaru, S. M. Reach, J. Motl, M. E. Ehrlich, Y. L. Hurd, P. Roussos, An atlas of chromatin accessibility in the adult human brain. *Genome Res.* **28**, 1243–1252 (2018).

57. A. Nott, I. R. Holtman, N. G. Coufal, J. C. M. Schlachetzki, M. Yu, R. Hu, C. Z. Han, M. Pena, J. Xiao, Y. Wu, Z. Keulen, M. P. Pasillas, C. O'Connor, C. K. Nickl, S. T. Schafer, Z. Shen, R.

A. Rissman, J. B. Brewer, D. Gosselin, D. D. Gonda, M. L. Levy, M. G. Rosenfeld, G. McVicker, F. H. Gage, B. Ren, C. K. Glass, Brain cell type-specific enhancer-promoter interactome maps and disease-risk association. *Science*. **366**, 1134–1139 (2019).

58. M. R. Corces, J. M. Granja, S. Shams, B. H. Louie, J. A. Seoane, W. Zhou, T. C. Silva, C. Groeneveld, C. K. Wong, S. W. Cho, A. T. Satpathy, M. R. Mumbach, K. A. Hoadley, A. G. Robertson, N. C. Sheffield, I. Felau, M. A. A. Castro, B. P. Berman, L. M. Staudt, J. C. Zenklusen, P. W. Laird, C. Curtis, Cancer Genome Atlas Analysis Network, W. J. Greenleaf, H. Y. Chang, The chromatin accessibility landscape of primary human cancers. *Science*. **362** (2018), doi:10.1126/science.aav1898.

59. Roadmap Epigenomics Consortium, A. Kundaje, W. Meuleman, J. Ernst, M. Bilenky, A. Yen, A. Heravi-Moussavi, P. Kheradpour, Z. Zhang, J. Wang, M. J. Ziller, V. Amin, J. W. Whitaker, M. D. Schultz, L. D. Ward, A. Sarkar, G. Quon, R. S. Sandstrom, M. L. Eaton, Y.-C. Wu, A. R. Pfenning, X. Wang, M. Claussnitzer, Y. Liu, C. Coarfa, R. A. Harris, N. Shoresh, C. B. Epstein, E. Gjoneska, D. Leung, W. Xie, R. D. Hawkins, R. Lister, C. Hong, P. Gascard, A. J. Mungall, R. Moore, E. Chuah, A. Tam, T. K. Canfield, R. S. Hansen, R. Kaul, P. J. Sabo, M. S. Bansal, A. Carles, J. R. Dixon, K.-H. Farh, S. Feizi, R. Karlic, A.-R. Kim, A. Kulkarni, D. Li, R. Lowdon, G. Elliott, T. R. Mercer, S. J. Neph, V. Onuchic, P. Polak, N. Rajagopal, P. Ray, R. C. Sallari, K. T. Siebenthall, N. A. Sinnott-Armstrong, M. Stevens, R. E. Thurman, J. Wu, B. Zhang, X. Zhou, A. E. Beaudet, L. A. Boyer, P. L. De Jager, P. J. Farnham, S. J. Fisher, D. Haussler, S. J. M. Jones, W. Li, M. A. Marra, M. T. McManus, S. Sunyaev, J. A. Thomson, T. D. Tlsty, L.-H. Tsai, W. Wang, R. A. Waterland, M. Q. Zhang, L. H. Chadwick, B. E. Bernstein, J. F. Costello, J. R. Ecker, M. Hirst, A. Meissner, A. Milosavljevic, B. Ren, J. A. Stamatoyannopoulos, T. Wang, M. Kellis, Integrative analysis of 111 reference human epigenomes. *Nature*. **518**, 317–330 (2015).

Supplementary Files

This is a list of supplementary files associated with this preprint. Click to download.

- TableS1.xlsx
- TableS2.xlsx
- TableS3.xlsx
- TableS4.xlsx
- TableS5.xlsx
- TableS6.xlsx
- TableS7.xlsx
